# miR-200a attenuated oxidative stress, inflammation, and apoptosis in dextran sulfate sodium-induced colitis through activation of Nrf2

**DOI:** 10.3389/fimmu.2023.1196065

**Published:** 2023-08-14

**Authors:** Shuai Peng, Lei Shen, Xiaoyun Yu, Jing Wu, Lanlan Zha, Yuan Xia, Hesheng Luo

**Affiliations:** ^1^ Department of Gastroenterology, Renmin Hospital of Wuhan University, Wuhan, China; ^2^ Hubei Key Laboratory of Digestive Diseases, Wuhan, China; ^3^ Department of Gastroenterology, Union Hospital, Tongji Medical College, Huazhong University of Science and Technology, Wuhan, China

**Keywords:** miR-200a, oxidative stress, inflammation, colitis, Nrf2

## Abstract

**Introduction:**

Oxidative stress and inflammatory responses are critical factors in ulcerative colitis disease pathogenesis. Nuclear factor erythroid 2-related factor 2 (Nrf2) modulates oxidative stress and suppresses inflammatory responses, and the protective benefits of Nrf2 activation have been associated with the therapy of ulcerative colitis. MicroRNA-200a (miR-200a) could target Kelch-like ECH-associated protein 1 (Keap1) and activate the Nrf2-regulated antioxidant pathway. Nevertheless, whether miR-200a modulates the Keap1/Nrf2 pathway in dextran sulfate sodium (DSS)-induced colonic damage is unknown. Here, our research intends to examine the impact of miR-200a in the model of DSS-induced colitis.

**Methods:**

Prior to DSS intervention, we overexpressed miR-200a in mice for four weeks using an adeno-associated viral (AAV) vector to address this problem. ELISA detected the concentration of inflammation-related cytokines. The genes involved in inflammatory reactions and oxidative stress were identified using quantitative reverse transcription-polymerase chain reaction (qRT-PCR), western blot, and immunofluorescence. Moreover, we applied siRNAs to weakened Nrf2 expression to confirm the hypothesis that miR-200a provided protection via Nrf2.

**Results:**

The present study discovered miR-200a down-regulation, excessive inflammatory activation, enterocyte apoptosis, colonic dysfunction, and Keap1/Nrf2 antioxidant pathway inactivation in mouse colitis and NCM460 cells under DSS induction. However, our data demonstrated that miR-200a overexpression represses Keap1 and activates the Nrf2 antioxidant pathway, thereby alleviating these adverse alterations in animal and cellular models. Significantly, following Nrf2 deficiency, we failed to observe the protective benefits of miR-200a against colonic damage.

**Discussion:**

Taken together, through activating the Keap1/Nrf2 signaling pathway, miR-200a protected against DSS-induced colonic damage. These studies offer an innovative therapeutic approach for ulcerative colitis.

## Graphical Abstract

miR-200a protected against DSS-induced colonic injury by activating the Keap1/Nrf2 signaling pathway. Overexpression of miR-200a alleviated intestinal inflammation, oxidative stress, apoptosis, and improved intestinal damage and pathological symptoms by silencing Keap1 and activating Nrf2, thereby protecting DSS-induced colonic injury. Significantly, following Nrf2 deficiency, we failed to observe the protective effects of miR-200a against DSS-induced colonic injury. These findings provide novel ideas and directions for treating ulcerative colitis.

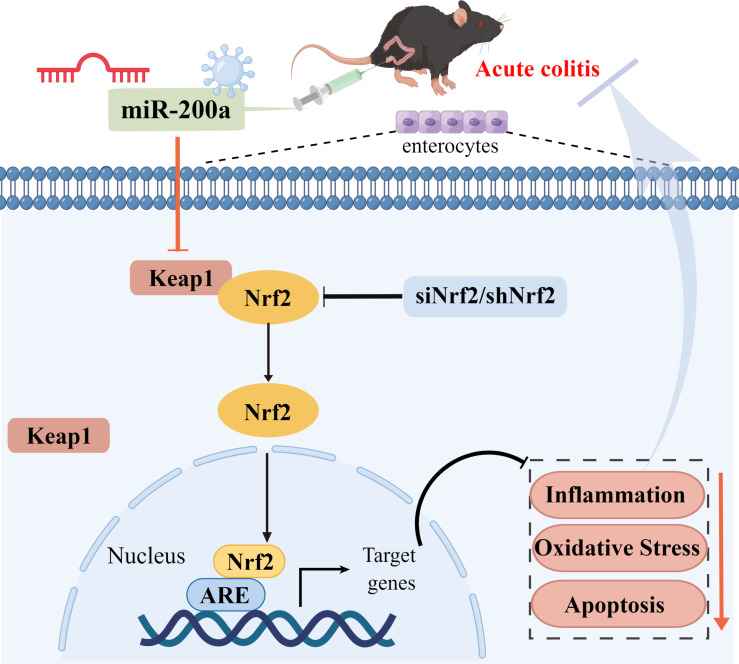

## 1 Introduction

Ulcerative colitis (UC) is a nonspecific, chronic, recurrent inflammatory disease of the colonic mucosa, characterized by colonic mucosal injuries and histological abnormalities (1). The underlying causes of ulcerative colitis are murky and complicated (2). There is mounting evidence that oxidative stress and inflammation are inextricably related to UC (3–5).

According to mounting evidence, the development of inflammation that causes UC may be aided by oxidative stress, which can affect disease progression in multiple ways (4, 6, 7). The inflammatory response generates reactive oxygen species (ROS) and reactive nitrogen species (RNS), destroying intestinal mucosal cells and structural changes in colon tissue (8, 9). Therefore, inhibition of these alterations would have significant implications for treating UC.

An essential component of antioxidant responses, Nrf2 controls the transcription of various detoxifying and antioxidant enzymes to protect against oxidative stress (10, 11). Under normal circumstances, Keap1 sequesters Nrf2 in the cytoplasm, rendering it inactive; As soon as cells experience oxidative stress, Nrf2 migrates to the nucleus and modulates the transcription of several antioxidant genes (11, 12). In DSS-induced colitis, Nrf2 expression was downregulated, and Nrf2 restoration might alleviate DSS-induced colonic damage (13, 14). These observations indicate that activation of Nrf2 might mitigate DSS-induced colitis.

MicroRNAs (miRNAs) are highly conserved single-stranded RNAs involved in various illnesses and pathological reactions, including UC (15, 16). At the same time, miRNAs can bind to the 3’-untranslated region (3’-UTR) of the target gene, causing protein expression to be degraded (17). Keap1/Nrf2 pathway activation has been demonstrated to be influenced by microRNAs, with the miR-200 family being especially crucial in maintaining the epithelial phenotype (18, 19). According to a recent study, miR-200a can target Keap1 mRNA, promoting Keap1 degradation, and leading to Nrf2 activation, thereby protecting mice from DOX-induced cardiotoxicity (20). Moreover, miR-200a can improve diabetic endothelial dysfunction by targeting Keap1/Nrf2 (21). In addition, enhancing miR-200a by Polydatatin to control the Keap1/Nrf2 pathway can prevent fructose-induced liver inflammation (22). These observations demonstrated that miR-200a could protect against colitis by regulating Nrf2. Nevertheless, whether miR-200a modulates the Keap1/Nrf2 pathway in DSS-induced colonic damage is unknown. The aim of this study was to investigate whether the alterations in the expression of miR-200a could impact the Keap1/Nrf2 pathways involved in the pathogenesis of colitis. We designed a mouse model supplemented with AAV-miR-200a and then induced colitis by administration of DSS for a week, followed by understanding the mechanisms that attenuate oxidative stress in colitis when overexpression of miR-200a. Here, we discovered that miR-200a activated Nrf2 by targeting Keap1, protecting mice from DSS-induced damage.

## 2 Materials and methods

### 2.1 Animals and treatment

C57BL/6 mice were purchased from Beijing Vital River Laboratory Animal Technology Co., Ltd. (Beijing, China). To overexpress miR-200a in the colon, male C57BL/6 mice, four weeks old and weighing 15-17g, received 1×10^12^ vg/mL of AAV2-miR-200a or AAV2-miR-scramble by clyster. Acute experimental colitis was generated after four weeks, apart from the control mice, by adding 3% DSS (30 mg/ml) to the water supply for seven days (n=10 for each group) (23). Colonic Nrf2 depletion was achieved by clyster of adenoviral vectors carrying Nrf2 small hairpin RNAs (shNrf2) or scrambled shRNA. Then, mice in the miR-scramble/miR-200a +DSS groups were administered 3%DSS (30 mg/ml) to mimic acute experimental colitis after the shNrf2 injection and the control mice received saline (n =10 for each group). During the experiment, each animal’s food and water intake and the disease activity index (DAI), which included body weights, stool consistency, and occult stool blood, were assessed daily (**Table 1**).

**Table 1:** Disease activity index score.

Score	Weight loss	Stool consistency	Bloody stool
0	none	normal	none
1	1-5%	paste stools	occult blood
2	6-10%	loose stools	bleeding
3	>10%	diarrhea	gross bleeding

### 2.2 Reagents

Dextran sulfate sodium (DSS; MW 36,000–50,000Da) was provided by MP Biomedicals (Aurora, OH, USA). Antibodies against Bcl-2 (A0208), Bax (A12009), Nrf2 (A1244), HO-1(A1346), Keap1 (A1820), NQO1(A0047) and GAPDH (AC027) were provided by Abclonal (Wuhan, China). Fluorescent antibodies against ZO-1 (61-7300) and Occludin (40-4700) were supplied by ThermoFisher Scientific (Waltham, USA). MicrON miR-200a and mimic were provided by Ribobio Technology (Guangzhou, China). The biochemical kits of ROS, SOD, MPO, MDA, 4-HNE, and GSH/GSSG were provided by Bioengineering Institute (Suzhou, China). TRIzol reagent was provided by Invitrogen (Carlsbad, CA). HiScript II Q Select RT SuperMix for qPCR Kit was provided by Vazyme Biotechnology (Nanjing, Suzhou, China). MTT Assay Kit was provided by Biosharp (Beijing, China). Annexin V-FITC Apoptosis Detection Kit was provided by KeyGEN Biotechnology (Nanjing, Suzhou, China). AAV2 and shNrf2 were provided by Hanbio Technology (Shanghai, China).

### 2.3 Histopathological assessment

All the colorectal and ileocecal parts of the mice were taken, and the colon length was assessed. Then, the dissected colon tissue was washed with cold phosphate-buffered saline (PBS). Part of the distal colon was cut and fixed with 4% paraformaldehyde for the histopathological analysis. The fixed intestinal tissues were embedded, sectioned, and stained using hematoxylin and eosin(H&E). The remaining colon was preserved in a refrigerator for other detection and quantification. Intestinal inflammation was evaluated blindly, and the histological score was calculated.

### 2.4 Enzyme-linked immunosorbent assay

The intestinal tissues were rinsed with pre-cooled PBS to remove residual blood, weighed and appropriate volume of intestinal tissues were sheared. Then, the sheared tissue was fully homogenized using a homogenizer after adding the appropriate volume of PBS and the supernatant was collected by centrifugation at 1500 g for 20 min at 4°C for further analysis. The inflammatory factors were measured using ELISA kits per the manufacturer’s instructions (MultiSciences Biotechnology, Hangzhou, Zhejiang, China).

### 2.5 Immunofluorescence assays

After dewaxing, antigen repair, and blocking, sections were incubated overnight with primary antibodies against ZO-1 (1:500) and Occludin (1:250), followed by the corresponding secondary antibodies. Finally, the nuclei were stained, and the sections were sealed. Images were captured under the OLYMPUS BX53 microscope with 20X or 40X objective.

### 2.6 Biochemical detection in colonic tissues

The fresh colonic tissue or cultured NCM460 cells were sufficiently lysed to extract the supernatant for quantitative analysis. ROS, SOD, MPO, MDA, 4-HNE, and GSH/GSSG activity levels in colonic tissues were analyzed using a relevant assay kit.

### 2.7 Cell culture

NCM460 colonocyte, a human colonic epithelial cell line, was transfected with micrON miR-200a/mimic for 48 h, followed by adding DSS (20 mg/ml) or PBS for 12 h. NCM460 colonocytes were transfected with siNrf2 (50 nmol/l) to suppress Nrf2 to confirm the hypothesis of this paper. An MTT assay kit assayed cell viability.

### 2.8 Flow cytometry

The NCM460 colonocyte was collected and centrifuged to remove the supernatant after the intervention. Then, the cells were intervened with AnnexinV-FITC/PI. Finally, the cells were collected after washing and analyzed by flow cytometry.

### 2.9 Quantitative Real-time Polymerase Chain Reaction (qRT-PCR)

First, total RNA was extracted from colon samples or cultured cells using the TRIzol reagent. After RNA was isolated, the extracted RNA was quality checked and quantified. Furthermore, a reverse transcription reaction was performed to obtain the template cDNA. Then, real-time fluorescence quantification was detected by the SYBR Green probe and ABI 7500 system. The expression of mRNAs in samples was normalized to an endogenous reference gene (GAPDH) relative to a calibrator. The miRNA level was detected using a HiScript II Q Select RT SuperMix for qPCR Kit. U6 was used as the internal control of miRNA (**Table 2**).

**Table 2:** Primer sequences used for qPCR.

Name	Primer	Sequence
GAPDH	Forward	5’-GAGATCCCTCCAAAATCAAGTG-3’
	Reverse	5’-GATGATCTTGAGGCTGTTGTCA-3’
U6	Forward	5’-CGCTTCGGCAGCACATATAC-3’
	Reverse	5’-AAATATGGAACGCTTCACGA-3’
miR-200a	Forward	5’-TGCGC TAACACTGTCTGGTAAC-3’
	Reverse	5’-CCAGTGCAGGGTCCGAGGTATT-3’
SOD1	Forward	5’-TGAAGGTGTGGGGAAGCATT-3’
	Reverse	5’-GTCACATTGCCCAAGTCTCC-3’
SOD2	Forward	5’-CCCGACCTGCCCTACGACTA-3’
	Reverse	5’-CTCCCCTTTGGGTTCTCCAC-3’
NQO1	Forward	5’-TGTGATATTCCAGTTCCCCC-3’
	Reverse	5’-AATGACATTCATGTCCCCGT-3’
HO-1	Forward	5’-TCTTTGAGGAGTTGCAGGAGC-3’
	Reverse	5’-AGTGTAAGGACCCATCGGAGAA-3’
Keap1	Forward	5’-CTGTTGAGGCACTTTTGTTTCT-3’
	Reverse	5’-CCCGCTTTGGACTTCTTTTGA-3’
Nrf2	Forward	5’- TTGCCTGTAAGTCCTGGTCA -3’
	Reverse	5’- CCCCTCCTACGTATATCCCG -3’

### 2.10 Western blot analysis

First, total protein from intestinal tissue or cultured colonocyte was extracted and centrifuged to collect lysates for protein quantification. The supernatant was denatured by heating it in boiling water. Electrophoresis was used to separate proteins, which were subsequently electrotransformed and blocked before being incubated with primary antibodies against Bcl-2, Bax, Nrf2, HO-1, NQO1, and Keap1, followed by the corresponding secondary antibodies. Finally, imaging analysis is performed after washing the bands in TBST.

### 2.11 TUNEL staining

According to the TUNEL apoptosis assay kit instructions (Vazyme Biotechnology, Nanjing, Suzhou, China), apoptotic cells in intestinal tissue and cultured colonocyte were detected. The nucleus was labeled with DAPI. The apoptotic cells were observed under a fluorescence microscope with 200 × or 400 × magnification and TUNEL-positive cells were counted. The images of TUNEL positive for quantification were analyzed using Image J software (NIH, Baltimore, MD, USA). Data were presented as a mean percentage of the number of TUNEL-positive cells in the whole fields of view from six randomly-selected fields in each per experiment.

### 2.12 Luciferase assay

First, Future Biotherapeutics constructed a target Keap1 dual luciferase reporter gene vector and a mutant with a mutation in the miR-200a binding site and transfected them into NCM460 cells. Next, micrON miR-200a and a negative control plasmid were transfected into Enterocytes. Then, the cells were lysed, and the supernatant was collected after 48 hours. Finally, luciferase activity was measured using the Promega assay system.

### 2.13 Statistical analyses

The results are presented as mean ± standard error of the mean (SEM), and all data were performed using SPSS 25.0 software. The differences between the two groups mean values were calculated using an unpaired t-test or nonparametric statistics. One-way ANOVA was employed to examine the differences between more than two groups, followed by the Tukey *post hoc* test. P-values less than 0.05 were deemed statistically significant.

## 3 Results

### 3.1 MiR-200a was decreased in DSS-induced colonic damage

**Figure 1:**
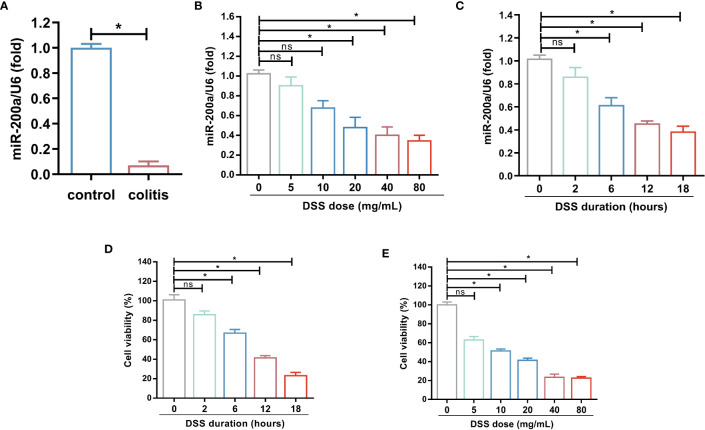
miR-200a was decreased in DSS-induced colonic damage. **(A)** The miR-200a expression in the colon of normal and DSS-induced colitis in mice for 7 days. **(B)** The miR-200a expression in NCM460 cells treated with various doses of DSS (doses ranging from 0-80 mg/mL) for 12h. **(C)** The miR-200a expression in NCM460 cells treated with 2% DSS at different times (0, 2, 6, 12, 18h). **(D)** Cell viability of NCM460 cells treated with 2% DSS at different times (0, 2, 6, 12, 18h). **(E)** Cell viability of NCM460 cells treated with various doses of DSS (doses ranging from 0-80 mg/mL) for 12h. Data are provided as mean ± SEM and reflect three experiments, analyzed using unpaired t-test or one-way ANOVA with Tukey *post-hoc* analysis. ^∗^P < 0.05; ns, not significant.

To explore miR-200a alterations in DSS-induced colonic damage, we evaluated its expression in the DSS-induced models. The colonic miR-200a levels were significantly decreased after the DSS intervention (**Figure 1A**). At the same time, DSS doses ranging from 0-80 mg/mL were given to the NCM460 cells. Moreover, we discovered that DSS treatment reduced the level of miR-200a in the NCM460 cells in a dose- and time-dependent manner (**Figures 1B, C**). Further study revealed that the cell viability of the NCM460 cells was reduced in a dose- and time-dependent manner (**Figures 1D, E**). When the induced dosage was greater than 20 mg/mL, the viability of the colonocyte was decreased to less than 50%. The preceding experimental data revealed that miR-200a might be crucial in colonic damage.

### 3.2 MiR-200a overexpression attenuates colonic damage in mice with DSS treatment

**Figure 2:**
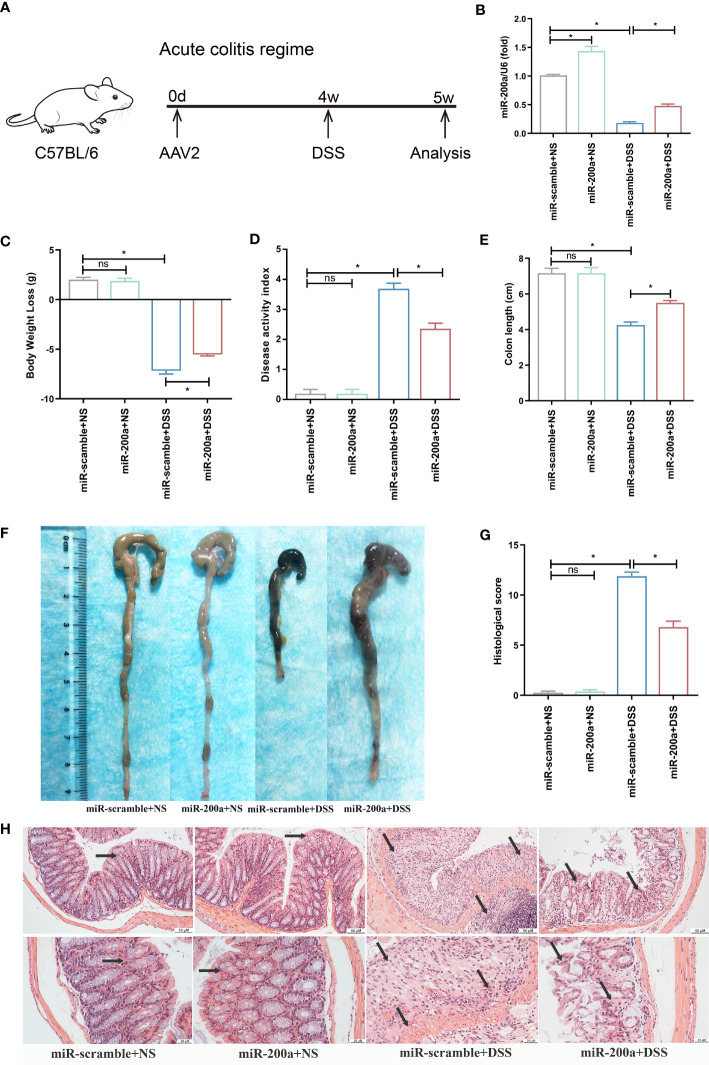
miR-200a overexpression attenuates colonic damage in mice with DSS treatment. **(A)** The timetable of the acute colitis regime trial. **(B)** The miR-200a expression in the colon after AAV2-miR-200a or AAV2-miR-scramble injection by clyster for 5 weeks. **(C)** The alteration in body weight of mice in the experiment period. **(D)** Disease activity index scores of mice in the experiment period. **(E, F)** Representative colon pictures of mice from different groups and the comparison of colon length. **(G)** Histological scores of mice from different groups. **(H)** Representative images of mice colon sections stained with H&E. (original magnifications, ×200, scale bars =50 μm; ×400, scale bars =20 μm). Data are provided as mean ± SEM and reflect six experiments, analyzed using one-way ANOVA with Tukey *post-hoc* analysis. ^∗^P < 0.05; ns, not significant.

Mice were first infected with AAV2 by clyster to overexpress miR-200a in the colon for 4 weeks before being given DSS or saline for seven days to recognize the role of miR-200a in experimental colitis (**Figure 2A**). Administration of DSS intervention reduced colonic miR-200a expression, which was restored in mice by injection of AAV2-miR-200a (**Figure 2B**). After seven days of DSS induced, miR-200a overexpression relieved the body weight decrease in colitis (**Figure 2C**). The DAI score was increased, and this alteration was reversed by AAV2-miR-200a injection in experimental colitis (**Figure 2D**). Meanwhile, shortening of the colon length was observed after DSS treatment, but miR-200a overexpression alleviated this reduction (**Figures 2E, F**). The colonic mucosal epithelial cells were intact structure, the lamina propria gland was normal, arranged neatly, and the crypts were normal in the mice with miR-200a or miR-scramble after saline treatment. In contrast, administration of DSS showed an obvious acute inflammatory reaction. Nevertheless, miR-200a overexpression reduced the colonic mucosa injury in mice. Please note that, after DSS treatment, the histological scores obtained with miR-200a overexpression were markedly lower than those obtained with a positive group (**Figures 2G, H**).

### 3.3 MiR-200a supplementation attenuated colonic inflammation accumulation and suppressed colonic apoptosis in DSS-treated mice

**Figure 3:**
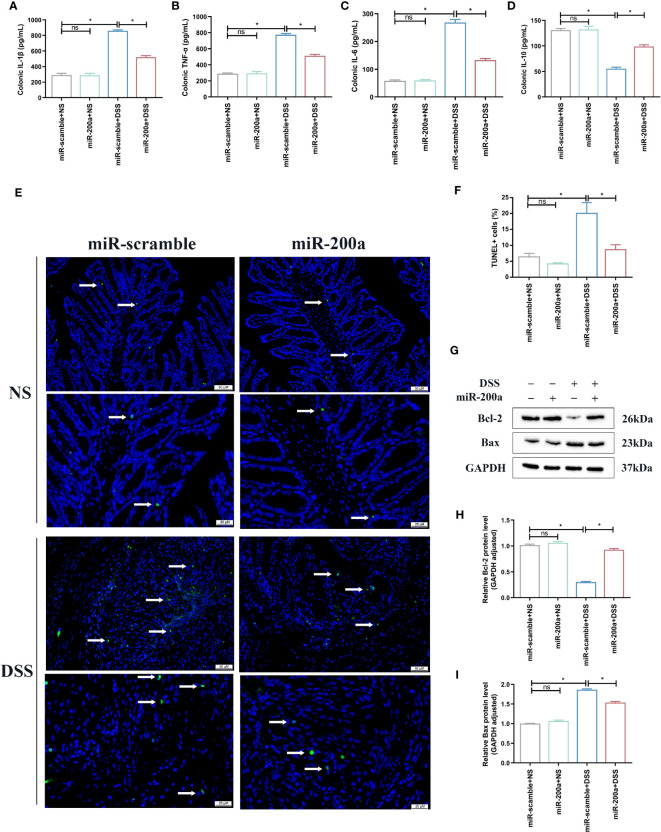
miR-200a supplementation attenuated colonic inflammation accumulation and suppressed colonic apoptosis in DSS-treated mice. **(A–D)** Colonic inflammatory factors (IL-1β, TNF-α, IL-6, IL-10) detected using ELISA kits. **(E, F)** Representative images of TUNEL staining in colon samples from different experimental groups. (Original magnifications, ×200, scale bars =50 μm; ×400, scale bars =20 μm). The images of TUNEL positive for quantification were analyzed using Image J software. **(G)** Representative immunoblot bands for the Bax and Bcl-2 proteins in the colon of mice. GAPDH was used as a loading control. **(H, I)** Histogram of relative expression of Bax and Bcl-2 proteins in the colon of mice. Data are provided as mean ± SEM and reflect six experiments, analyzed using one-way ANOVA with Tukey *post-hoc* analysis. ^∗^P < 0.05; ns, not significant.

First, ELISA detected the concentration of inflammatory cytokines in the intestine. The colon’s TNF-α, IL-6, and IL-1β expressions were raised in experimental colitis. These rises, however, were mitigated in mice following miR-200a overexpression (**Figures 3A–C**). The expression of IL-10 was reduced in colitis, and AAV2-miR-200a supplementation corrected the drop (**Figure 3D**). In addition, in DSS-induced colitis, the percentage of apoptotic cells was much higher compared to the mice treated with saline. However, this situation was alleviated after miR-200a overexpression in mice (**Figures 3E, F**). Subsequent western analysis demonstrated that miR-200a boosted the expression of Bcl-2 and suppressed the expression of Bax in mice exposed to DSS (**Figures 3G–I**).

### 3.4 MiR-200a overexpression attenuated oxidative stress and protected against barrier disruption in DSS- induced mice

**Figure 4:**
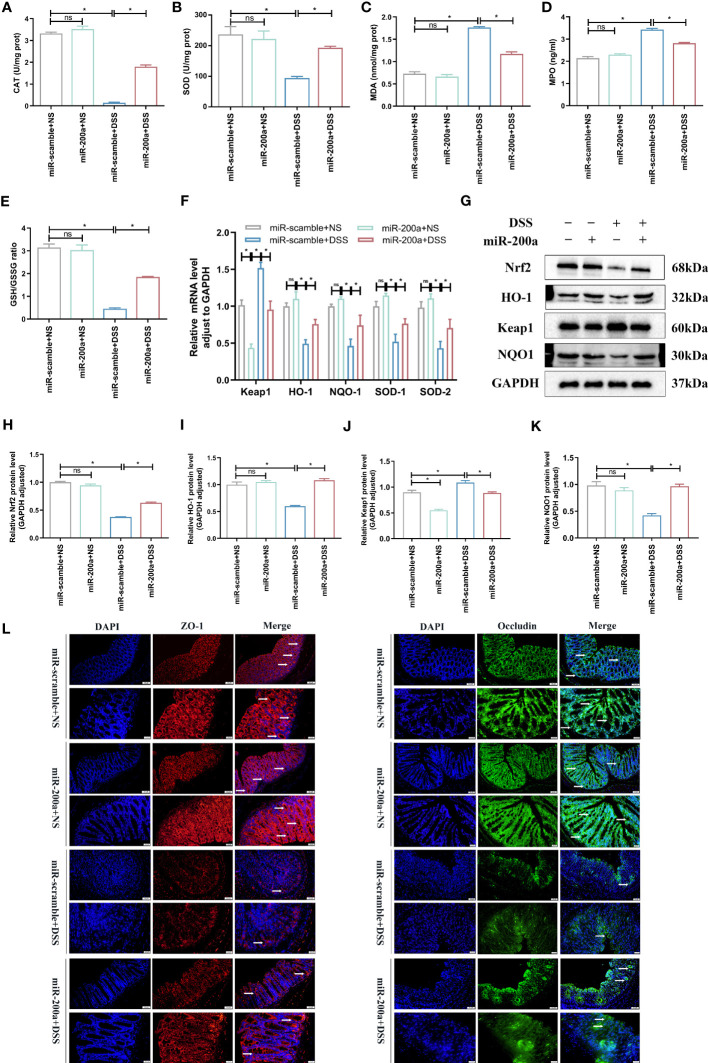
miR-200a overexpression reduced oxidative stress and protected against barrier disruption in DSS-induced mice. **(A, B)** The level of CAT and the activity of SOD in colon samples of mice were detected by commercial kits. **(C, D)** The level of MDA and the activity of MPO in colon samples of mice were detected by commercial kits. **(E)** The ratio of GSH/GSSG in colon samples of mice was detected by commercial kits. **(F)** The mRNA expression of the Nrf2-regulated gene was detected by qRT-PCR in mice. **(G)** Representative immunoblot bands for the Nrf2, HO-1, Keap1, and NQO1 proteins in the colon of mice. GAPDH was used as a loading control. **(H–K)** Histogram of relative expression of Nrf2, HO-1, Keap1 and NQO1 proteins in the colon of mice. **(L)** Representative images of immunofluorescence staining of ZO-1(red) and Occludin (green) in colon samples from different experimental groups. (Original magnifications, ×200, scale bars =50 μm; ×400, scale bars =20 μm). Data are provided as mean ± SEM and reflect six experiments, analyzed using one-way ANOVA with Tukey *post-hoc* analysis. ^∗^P < 0.05; ns, not significant.

The colonic oxidative stress-related indicators were detected in mice. The CAT and SOD activities were reduced in colitis, and over-expression of miR-200a prevented this downregulation (**Figures 4A, B**). MDA and MPO activities, on the other hand, accumulated abnormally in DSS-induced colitis; however, this situation was suppressed after miR-200a intervention (**Figures 4C, D**). Simultaneously, the ratio of GSH/GSSG was decreased in DSS-induced mice, and this condition was inhibited after miR-200a over-expression in mice (**Figure 4E**). Furthermore, several essential Nrf2-dependent genes were detected in the colon. The mRNA expression of HO-1, NQO-1, SOD-1, and SOD-2 was restored in the colon after miR-200a overexpression, whereas the expression of Keap1 was suppressed (**Figure 4F**). Further results confirmed that Nrf2, HO-1, and NQO1 were reduced in DSS-induced mice, whereas Keap1 was enhanced. However, miR-200a restored the decreased Nrf2, HO-1, and NQO1 protein expression and reduced the increased Keap1 protein expression in the DSS-induced colon (**Figures 4G–K**). At the same time, the loss of TJ proteins is the key factor contributing to barrier disruption. As shown in **Figure 4L**, the expression of ZO-1 and Occluding were decreased in colitis, and those barrier disruptions were restored by miR-200a overexpression (**Figure 4L**).

### 3.5 MiR-200a overexpression attenuated DSS-induced colonic injury *in vitro*

**Figure 5:**
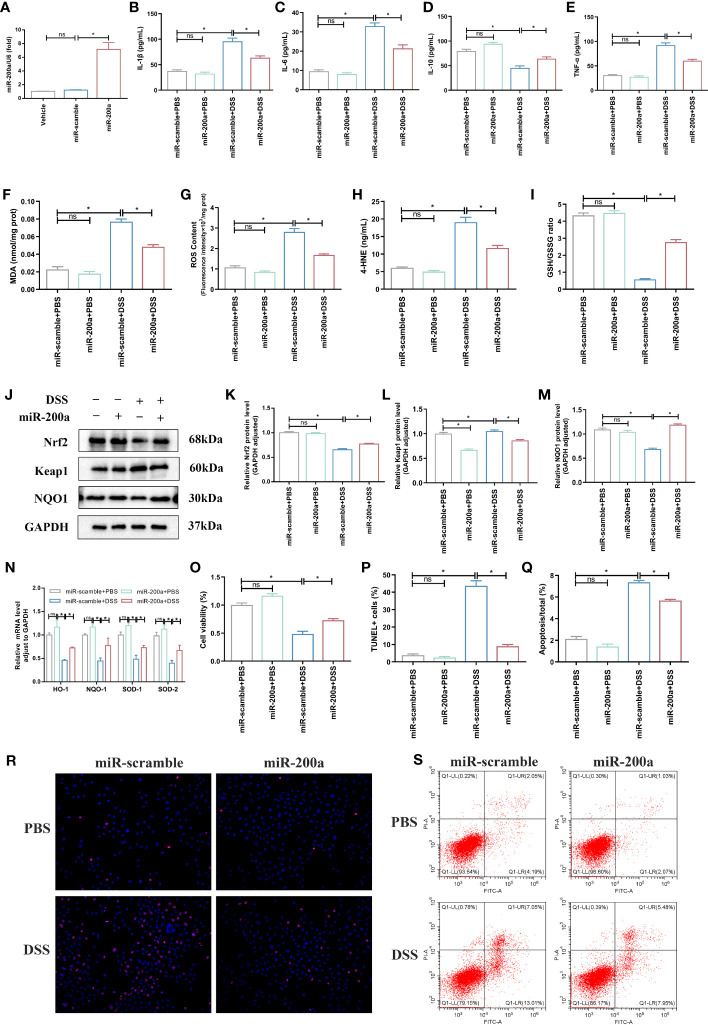
miR-200a overexpression attenuated DSS-induced colonic injury *in vitro*. **(A)** The expression of miR-200a in cells transfected with the miR-200a mimic or the miR-scramble control for 48 h. **(B–E)** The levels of inflammatory cytokines (IL-1β, TNF-α, IL-6, IL-10) were analyzed by ELISA kits in the NCM460 cells. **(F)** The production of MDA in Enterocytes was detected by commercial kits. **(G)** The activity of ROS in Enterocytes was detected by commercial kits. **(H, I)** The expression of 4-HNE and GSH/GSSG in Enterocytes was detected by commercial kits. **(J–M)** Representative immunoblot bands for the Nrf2, Keap1, and NQO1 proteins in Enterocytes. GAPDH was used as a loading control. **(N)** The mRNA levels of the Nrf2-regulated gene were detected by qRT-PCR in the NCM460 cells. **(O)** Cell viability detected by MTT in Enterocytes from different experimental groups. **(P, R)** Representative images of TUNEL staining in the NCM460 cells from different experimental groups. (Original magnifications, ×200, scale bars =50 μm);. The images of TUNEL positive for quantification were analyzed using Image J software. **(Q, S)** Representative images of the percentage of apoptosis were examined by flow cytometry. Data are provided as mean ± SEM and reflect three experiments, analyzed using one-way ANOVA with Tukey *post-hoc* analysis. ^∗^P < 0.05; ns, not significant.

Compared to the control group, the miR-200a overexpression group significantly ameliorated DSS-induced inflammation accumulation in NCM460 cells (**Figures 5A–E**). At the same time, the supplementation of miR-200a reduced the expression of MDA, 4-HNE, and ROS in DSS-induced NCM460 cells (**Figures 5F–H**). In contrast, the GSH/GSSG ratio decreased in DSS-induced cells, which was suppressed after overexpression of miR-200a in the colonocyte (**Figure 5I**). Furthermore, the protein expression of Nrf2 and NQO1 was reduced, and Keap1 was raised in DSS-induced Enterocytes. However, miR-200a restored the decreased Nrf2 and NQO1 protein expression and reduced the increased Keap1 protein expression in the DSS-induced Enterocytes (**Figures 5J–M**). Moreover, several essential Nrf2-dependent genes were detected in NCM460 cells. The mRNA expression of HO-1, NQO-1, SOD-1, and SOD-2 was restored in the NCM460 cells after miR-200a overexpression (**Figure 5N**). Moreover, miR-200a overexpression improved the impaired enterocyte viability (**Figure 5O**). In addition, the proportion of TUNEL-positive and apoptosis cells in DSS-induced NCM460 cells was significantly increased. However, miR-200a treatment reduced the DSS-induced TUNEL-positive and apoptotic cells (**Figures 5P–S**).

### 3.6 MiR-200a provided colonic protection via activation of Nrf2

**Figure 6:**
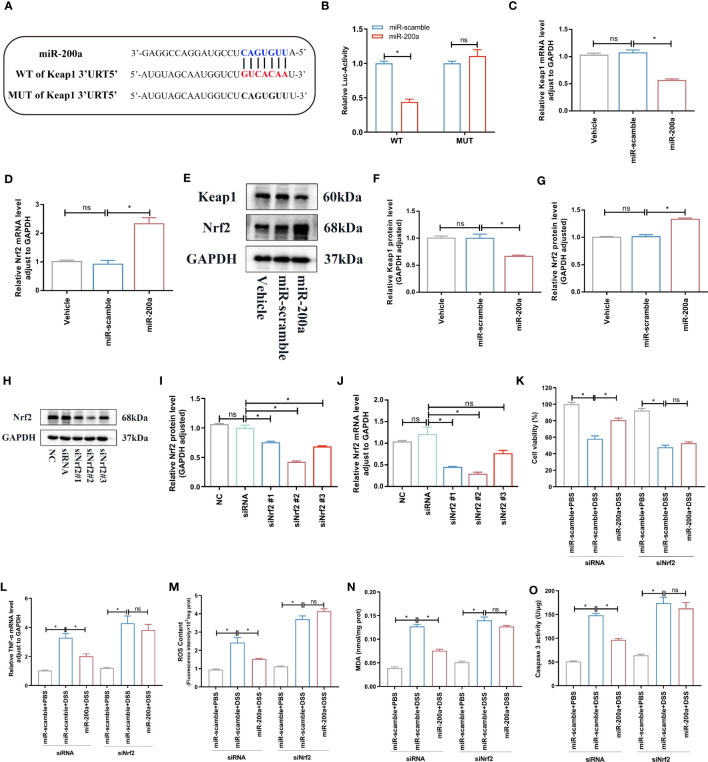
miR-200a targeted Keap1 and activated Nrf2. **(A)** Predicted miR-200a binding sites in the Keap1 3’-UTR. **(B)** Dual-luciferase reporter gene assay. **(C, D)** The mRNA levels of Keap1 and Nrf2 detected by qRT-PCR in the Enterocytes. **(E)** Representative immunoblot bands for the Nrf2 and Keap1 proteins in the NCM460 cells. GAPDH was used as a loading control. **(F–G)** Histogram of relative expression of Nrf2 and Keap1 proteins in in the NCM460 cells. **(H–J)** The expression of Nrf2 was tested by qRT-PCR and western blotting in cells. **(K)** Cell viability detected by MTT in Enterocytes from different experimental groups. **(L)** The TNF-α mRNA level detected by qRT-PCR in Enterocytes. **(M, N)** The ROS and MDA production in the NCM460 cells. **(O)** The caspase 3 activity in the NCM460 cells. Data are provided as mean ± SEM and reflect three experiments, analyzed using one-way ANOVA with Tukey *post-hoc* analysis. ^∗^P < 0.05; ns, not significant. UTR, untranslated region; WT, wild type; MUT, mutant.

Luciferase assays revealed that miR-200a could target the 3′-UTR of Keap1, and cells overexpressing miR-200a lowered the level of Keap1 mRNA (**Figures 6A–C**). Moreover, we discovered that the miR-200a treatment boosted the expression of the Nrf2 mRNA (**Figure 6D**). Further results indicated that, in NCM460 cells, miR-200a restored the decreased Nrf2 protein expression and reduced the increased Keap1 protein expression (**Figures 6E–G**). Then, to prove that miR-200a conferred protection through Nrf2, we utilized siRNAs to suppress the Nrf2 level. The siNrf2#2 group markedly downregulated endogenous Nrf2 expression in PCR, and western analysis was employed in further research (**Figures 6H–J**). In DSS-induced cells, miR-200a dramatically increased cell survival and lowered caspase 3 activity, ROS content, TNF-α level, and MDA generation, and these improvements were lost following Nrf2 deficiency (**Figures 6K–O**).

**Figure 7:**
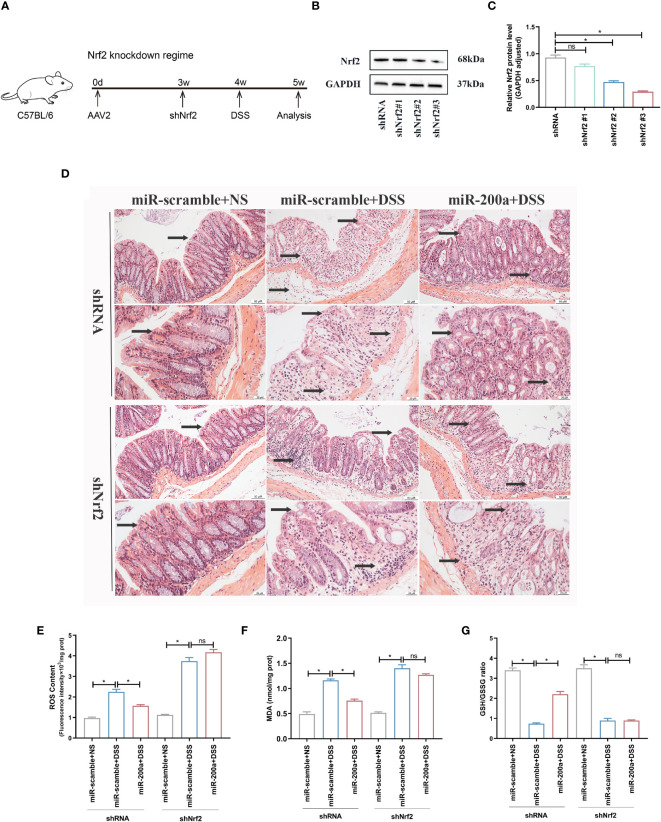
miR-200a could not provide colonic protection against DSS-induced acute colitis in Nrf2-deficient mice. **(A)** The timetable of the Nrf2 knockdown regime. **(B, C)** The protein expression of Nrf2 was detected in the colon after shRNA or shNrf2 treatment. GAPDH was used as a loading control. **(D)** Representative images of mice colon sections stained with H&E. (original magnifications, ×200, scale bars =50 μm; ×400, scale bars =20 μm). **(E, F)** The level of ROS and the activity of MDA in colon of shRNA or shNrf2 treated mice were detected by commercial kits. **(G)** The GSH/GSSG ratio in colon of shRNA or shNrf2 treated mice were detected by commercial kits. Data are provided as mean ± SEM and reflect six experiments, analyzed using one-way ANOVA with Tukey *post-hoc* analysis. ^∗^P < 0.05; ns, not significant.

We knocked down colonic Nrf2 to validate if miR-200a exerts its protective function by activating Nrf2 (**Figures 7A–C**). As expected, miR-200a lost its protective function in colonic damage, as indicated by GSH/GSSG ratio, ROS content, MDA production, and HE images in mice (**Figures 7D–G**).

## 4 Discussion

The expression and potential function of miR-200a in DSS-induced colonic damage have not been elucidated. Prior studies have noted the abnormal expression of miR-200a in UC, but the underlying mechanism has not been revealed (24–27). A previous study has revealed that miR-200a can protect intestinal epithelial cells by targeting RIPK1 (28). In our research, for the first time, we demonstrated that the expression of miR-200a was decreased in DSS-induced colitis, and supplementation of miR-200a protected mice and colonic epithelial cells from DSS-induced intestinal damage. It should be noted that miR-200a could improve colonic damage and alleviate colonic epithelial inflammation, oxidative stress damage, and apoptosis. These results further strengthened our confidence that miR-200a supplementation may be an effective method of preventing DSS-induced colitis.

It is widely acknowledged that oxidative stress and inflammation play crucial roles in the pathogenesis of UC (29). The two facilitate each other, further aggravating the structure and function of the intestine in colitis (30). Experimental mice suffered body weight loss, increased DAI score, shortened colon length, and bloody stools after administration of DSS. Furthermore, HE staining results showed that DSS-induced colonic mucosa exhibited obvious acute inflammatory response and intestinal structural disorder. However, our results demonstrated that miR-200a overexpression relieved the clinical manifestations and pathological changes in DSS-induced experimental colitis.

Excessive inflammation and an uncontrolled immune system led to an imbalance of oxidative and antioxidant systems in DSS-induced colitis (31, 32). Our current work revealed that pro-inflammatory factors were elevated, whereas antioxidants were decreased in DSS-induced mouse intestinal tissue and colonic epithelial cells. Nevertheless, miR-200a supplementation attenuated these changes, suggesting that its protective effects can be partially achieved by ameliorating oxidative stress damage.

In addition, oxidative stress is closely related to apoptosis (33). When excessive inflammation, an uncontrolled immune system, and continuous oxidative stress accumulate, intestinal epithelial cell apoptosis increases, disrupting intestinal mucosal integrity and barrier function (13, 34). In DSS-induced colitis, elevated pro-apoptotic protein Bax and reduced anti-apoptotic component Bcl-2 were seen in acute inflammatory areas. However, miR-200a supplementation reduced the apoptosis in DSS-induced injury. These findings illustrated that miR-200a supplementation relieved the pathological changes of colonic injury by attenuating intestinal inflammation, oxidative stress, and apoptosis.

Nowadays, Nrf2 is intimately implicated in developing DSS-induced colonic injury, and the protective effects of Nrf2 activation have been identified in the treatment of UC (34, 35). It is widely accepted that Keap1/Nrf2 signaling pathway is critical in coordinating antioxidant response mechanisms (36, 37). In UC, the Keap1/Nrf2 pathway can reduce intestinal inflammation and injury by controlling oxidative stress and play an essential role in protecting intestinal integrity, mainly by regulating inflammatory mediators and inducing the production of antioxidant enzymes (38). Oxidative stress provokes Nrf2 to translocate to the nucleus, where it mediates the transcription of a diverse suite of antioxidant genes, thereby conferring cell protection against the damage induced by oxidative stress (39). Mounting evidence has confirmed that the expression and activity of Nrf2 are reduced in DSS-induced colitis, and restoring Nrf2 by various drugs alleviated DSS-induced injury (35, 40, 41). Currently, most studies focus on regulating inflammatory mediators, inducing the production of antioxidant enzymes, and regulating autophagy by activating Nrf2, thereby reducing oxidative stress damage caused by aggravated ROS and alleviating pathological inflammatory responses (42, 43). Moreover, according to relevant studies, miRNA targeting Keap1 silencing is a new method to induce Nrf2 activation (44, 45). miR-152 exerts cardiac protection by targeting and silencing Keap1 to activate Nrf2 signaling (46). miR-141 inhibits vascular smooth muscle cell proliferation and migration by targeting the Keap1-Nrf2-HO-1 axis (47). miR-626 targeted Keap1 and activated Nrf2 to protect retinal pigment epithelial cells from oxidative damage (48).

In our study, luciferase assays revealed that miR-200a could target the 3′-UTR of Keap1. Further experimental outcomes revealed that miR-200a overexpression could degrade Keap1 and activate Nrf2 to suppress intestinal inflammation and oxidative stress. Moreover, activating various antioxidant genes downstream of the Keap1-Nrf2 signaling pathway, such as NQO-1, HO-1, and SOD, strengthens the defense against oxidative stress. Interestingly, once the Nrf2 expression was depleted, the potential regulation of the miR-200a in oxidative stress and inflammatory responses was eliminated. Therefore, we confirmed that Nrf2 activation is necessary for miR-200a to exert its protection.

In summary, our research demonstrated that miR-200a alleviated intestinal inflammation, oxidative stress, and apoptosis and improved intestinal function and pathological symptoms by silencing Keap1 and activating Nrf2, thereby protecting DSS-induced colonic injury. These findings provide novel ideas and directions for treating UC.

## Data availability statement

The original contributions presented in the study are included in the article/**Supplementary Material**, further inquiries can be directed to the corresponding author/s.

## Ethics statement

The Ethical Committee of Renmin Hospital of Wuhan University reviewed and approved the animal study protocol (WDRM-20211107A).

## Author contributions

SP and HL conceived and designed the study. SP, XY, JW, LZ, and YX performed the experiments. SP wrote the paper. SP, LS, and HL reviewed and edited the manuscript. All authors contributed to the article and approved the submitted version.
