# Supplementary material for: miR-200a attenuated oxidative stress, inflammation, and apoptosis in dextran sulfate sodium-induced colitis through activation of Nrf2
Source: Front Immunol. 2023 Aug 14;14:1196065. doi: 10.3389/fimmu.2023.1196065 (PMC10461398; doi:10.3389/fimmu.2023.1196065)

## Slide 1
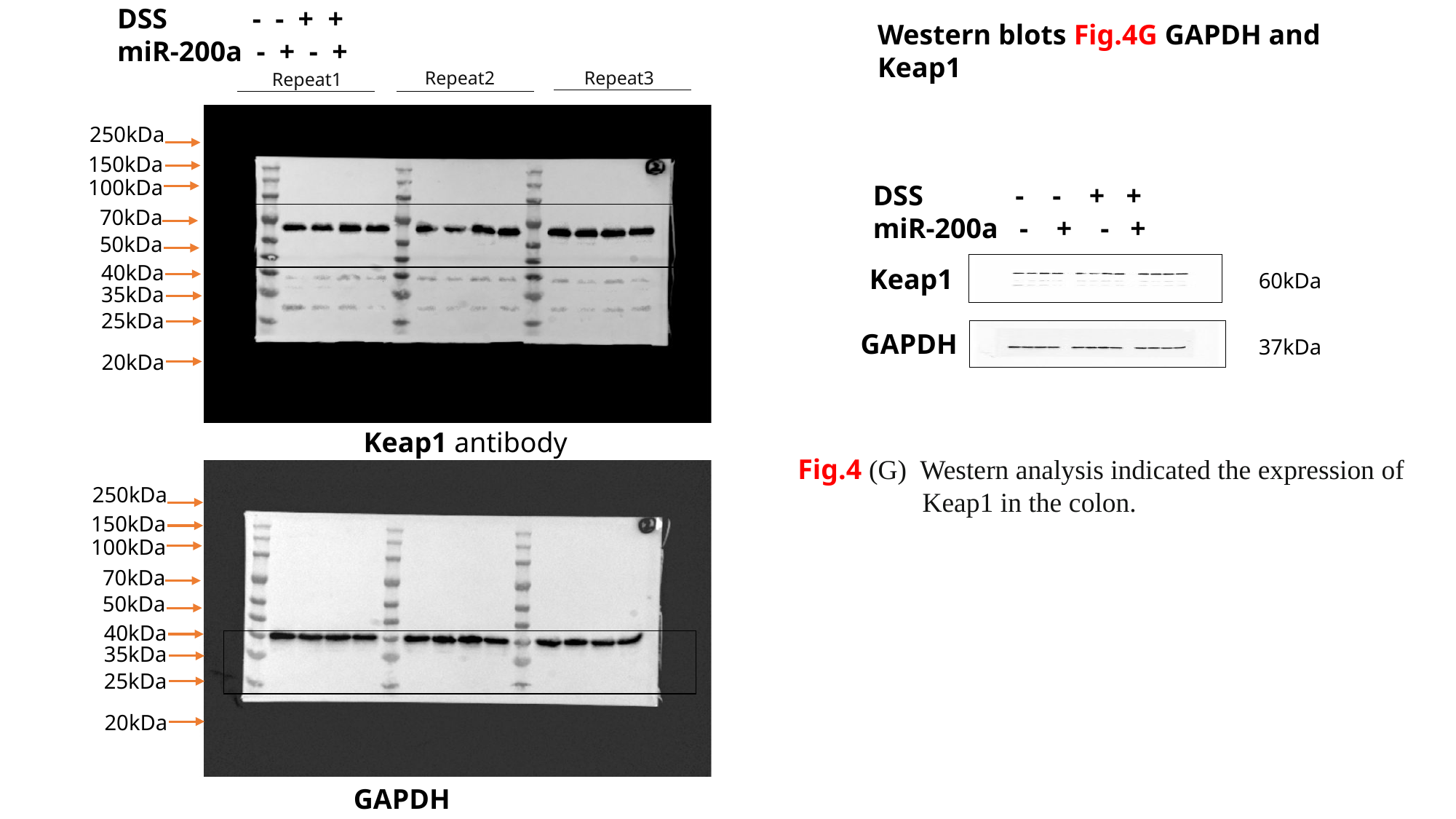

DSS - - + +
miR-200a - + - +
Western blots Fig.4G GAPDH and Keap1
Repeat2
Repeat3
Repeat1
250kDa
150kDa
100kDa
DSS - - + +
miR-200a - + - +
70kDa
50kDa
40kDa
Keap1
60kDa
35kDa
25kDa
GAPDH
37kDa
20kDa
Keap1 antibody
Fig.4 (G) Western analysis indicated the expression of
 Keap1 in the colon.
250kDa
150kDa
100kDa
70kDa
50kDa
40kDa
35kDa
25kDa
20kDa
GAPDH antibody

## Slide 2
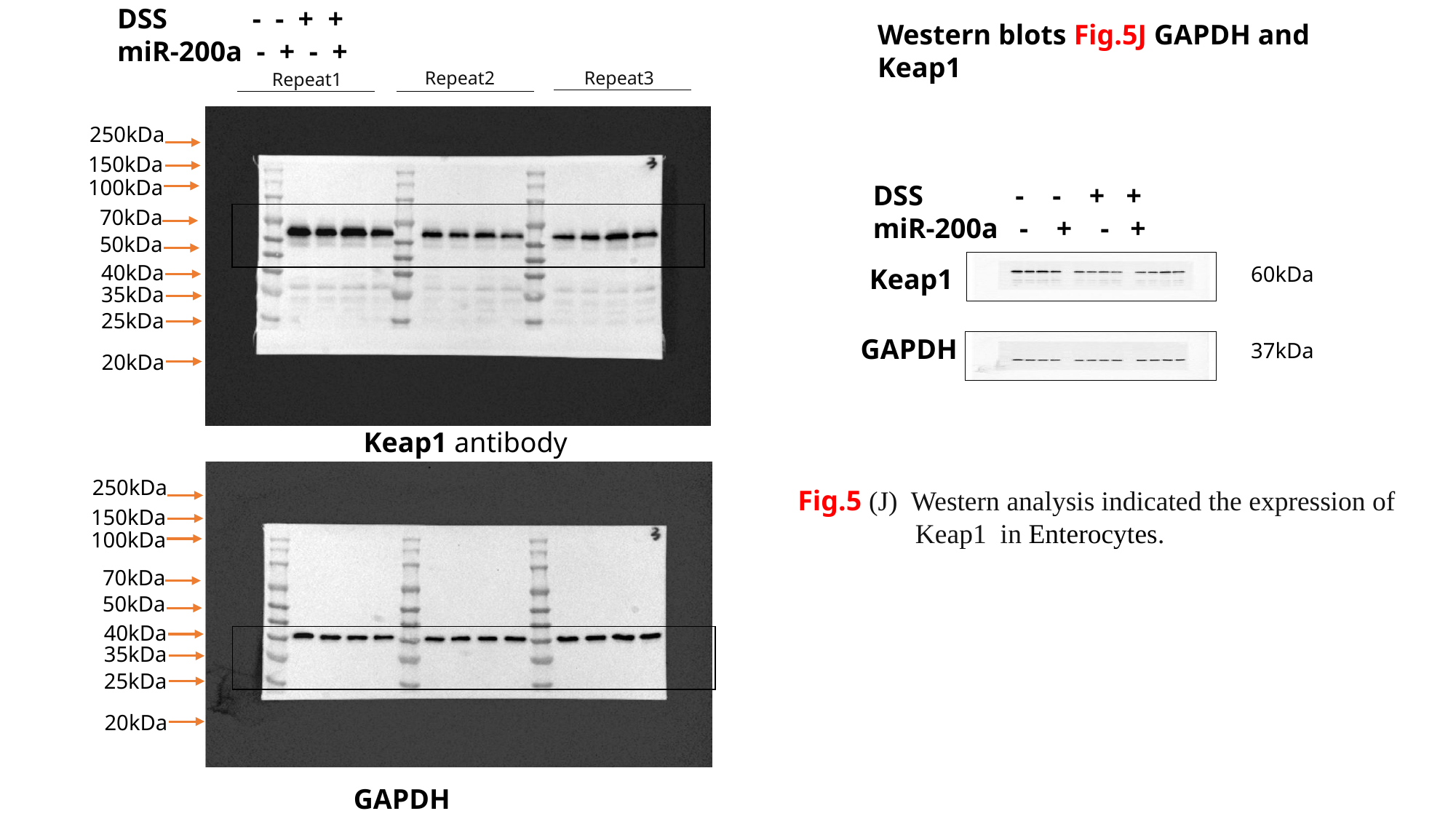

DSS - - + +
miR-200a - + - +
Western blots Fig.5J GAPDH and Keap1
Repeat2
Repeat3
Repeat1
250kDa
150kDa
100kDa
DSS - - + +
miR-200a - + - +
70kDa
50kDa
40kDa
60kDa
Keap1
35kDa
25kDa
GAPDH
37kDa
20kDa
Keap1 antibody
250kDa
Fig.5 (J) Western analysis indicated the expression of
 Keap1 in Enterocytes.
150kDa
100kDa
70kDa
50kDa
40kDa
35kDa
25kDa
20kDa
GAPDH antibody

## Slide 3
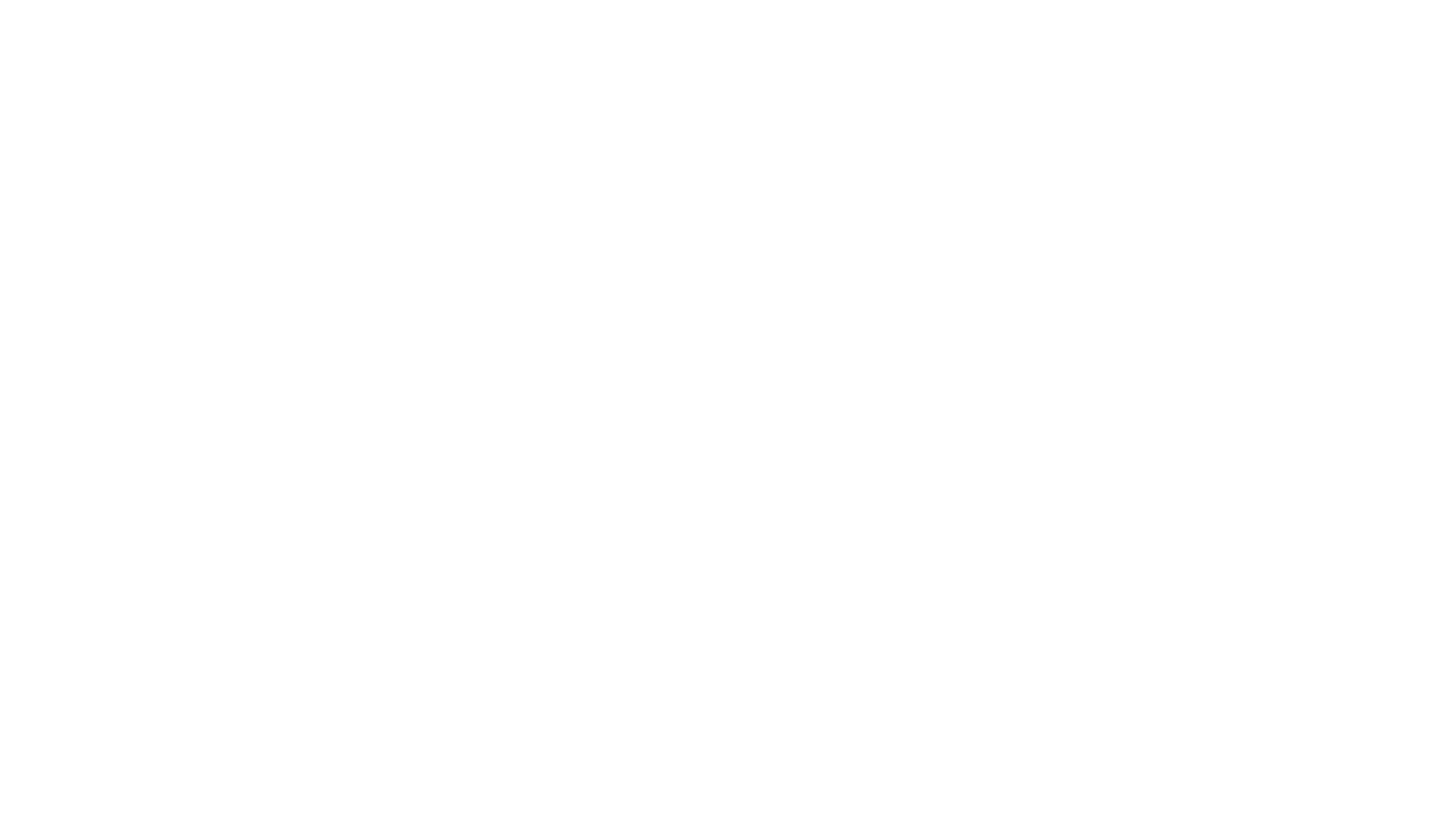

Supplement: Supplementary file 1 [file DataSheet_1.zip › Supplementary data/Western blot Fig4G and Fig5J-Keap 1-GAPDH.pptx]
