# Supplementary figures and images for: miR-200a attenuated oxidative stress, inflammation, and apoptosis in dextran sulfate sodium-induced colitis through activation of Nrf2
Source: Front Immunol. 2023 Aug 14;14:1196065. doi: 10.3389/fimmu.2023.1196065 (PMC10461398; doi:10.3389/fimmu.2023.1196065)

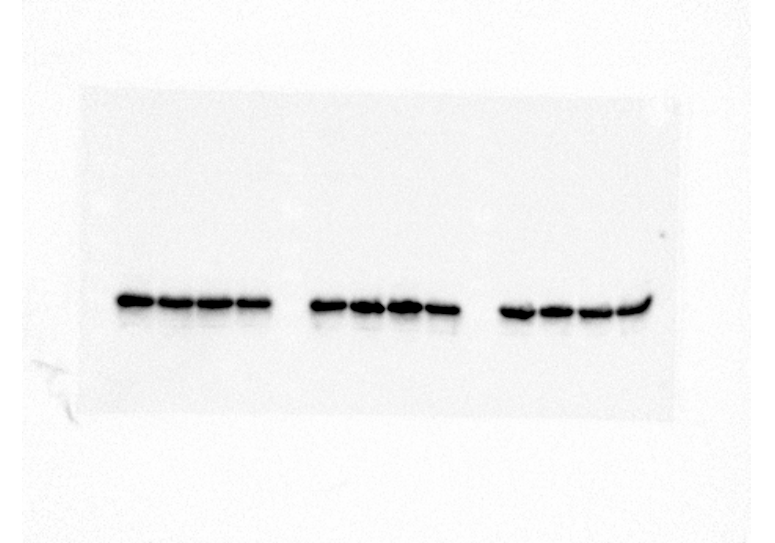

Supplement: Supplementary file 1 [file DataSheet_1.zip › Supplementary data/Figure4G/GAPDH-2-4G/GAPDH-2-D.tif]

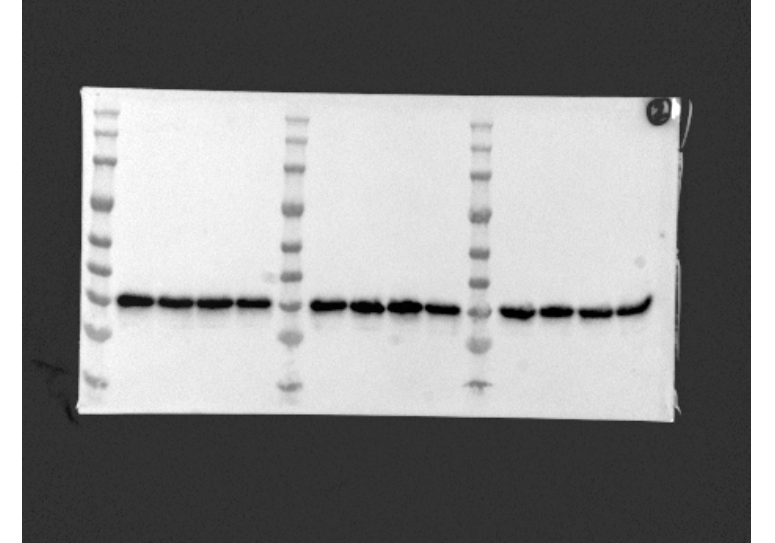

Supplement: Supplementary file 1 [file DataSheet_1.zip › Supplementary data/Figure4G/GAPDH-2-4G/MAKER-2-D+GAPDH-2-D.tif]

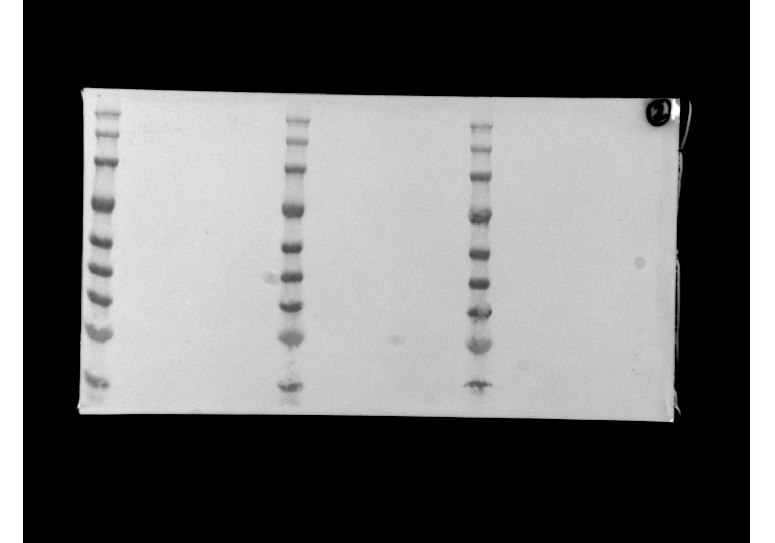

Supplement: Supplementary file 1 [file DataSheet_1.zip › Supplementary data/Figure4G/GAPDH-2-4G/MAKER-2-D.tif]

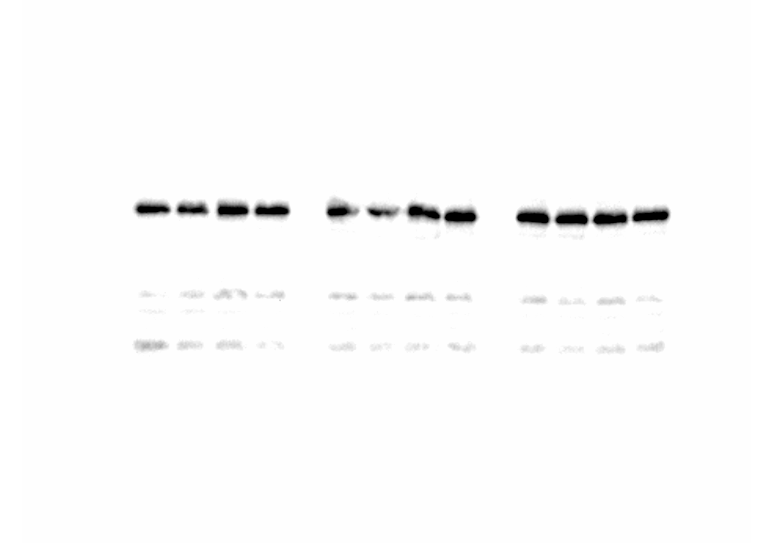

Supplement: Supplementary file 1 [file DataSheet_1.zip › Supplementary data/Figure4G/KEAP1-2-4G/KEAP1-2-D.tif]

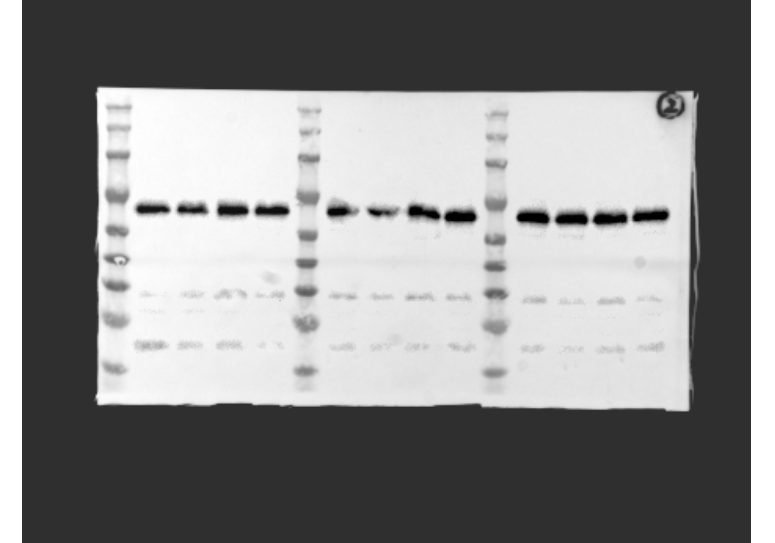

Supplement: Supplementary file 1 [file DataSheet_1.zip › Supplementary data/Figure4G/KEAP1-2-4G/MAKER-2-D-K+KEAP1-2-D.tif]

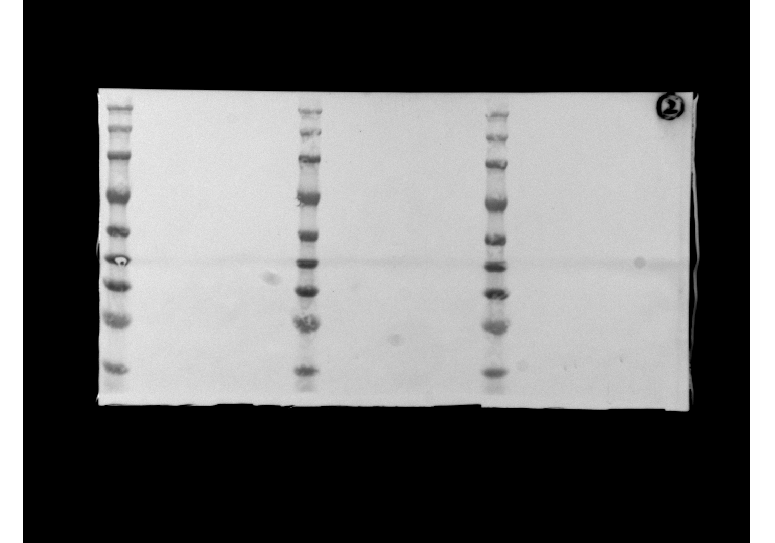

Supplement: Supplementary file 1 [file DataSheet_1.zip › Supplementary data/Figure4G/KEAP1-2-4G/MAKER-2-D-K.tif]

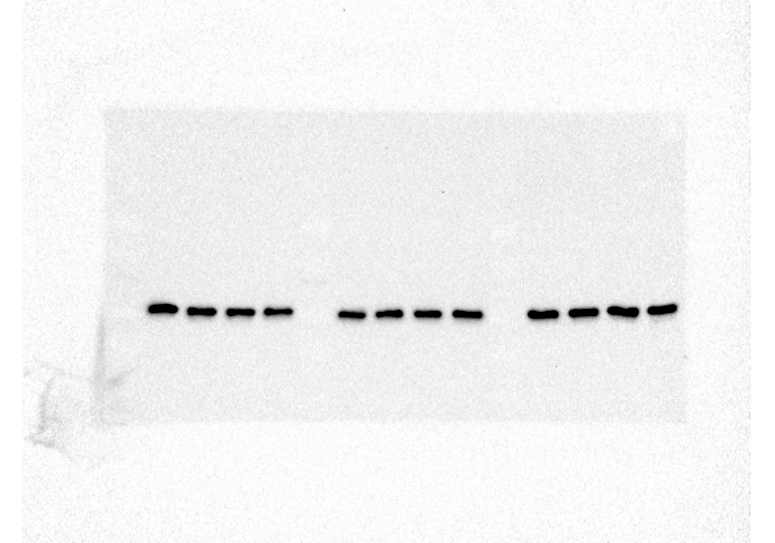

Supplement: Supplementary file 1 [file DataSheet_1.zip › Supplementary data/Figure5J/GAPDH-3-5J/GAPDH-3-C.tif]

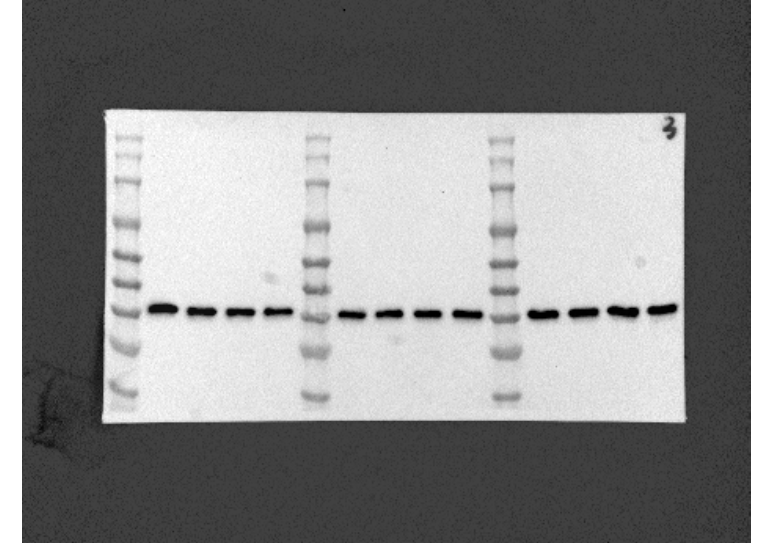

Supplement: Supplementary file 1 [file DataSheet_1.zip › Supplementary data/Figure5J/GAPDH-3-5J/MAKER-3-C+GAPDH-3-C.tif]

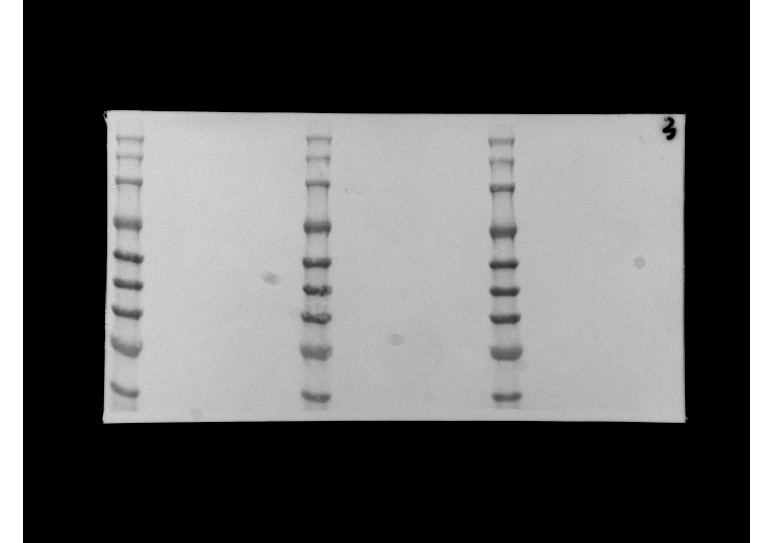

Supplement: Supplementary file 1 [file DataSheet_1.zip › Supplementary data/Figure5J/GAPDH-3-5J/MAKER-3-C.tif]

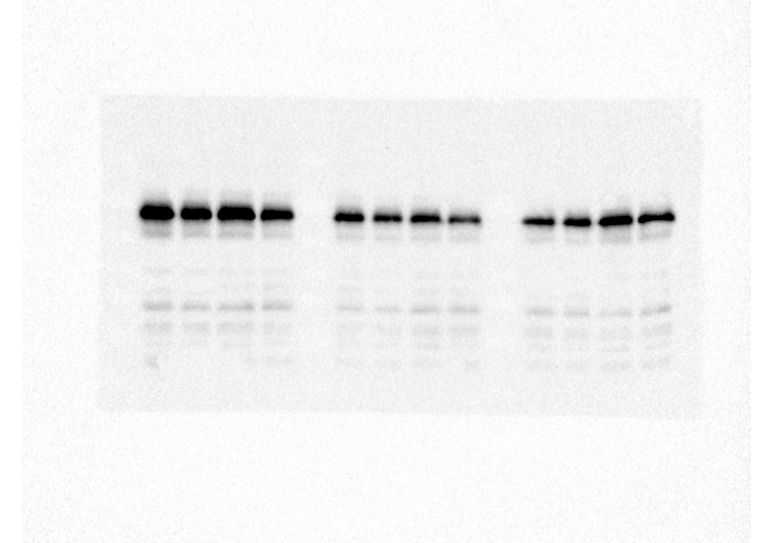

Supplement: Supplementary file 1 [file DataSheet_1.zip › Supplementary data/Figure5J/KEAP1-3-5J/KEAP-3-C.tif]

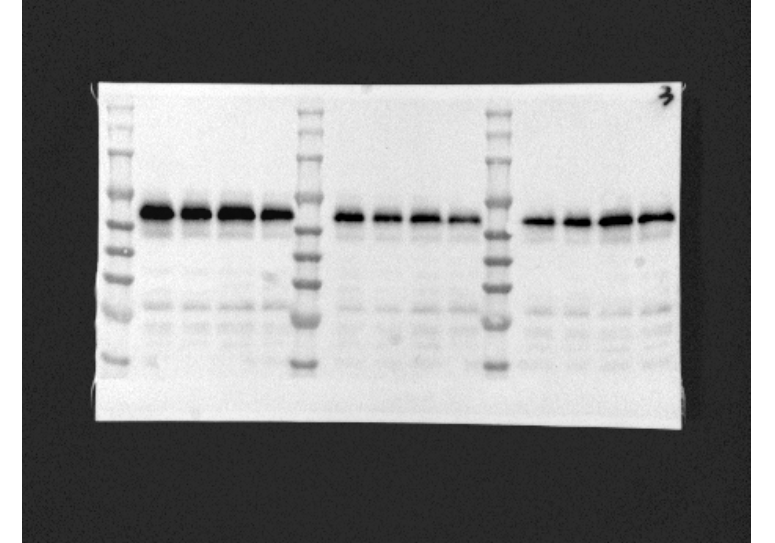

Supplement: Supplementary file 1 [file DataSheet_1.zip › Supplementary data/Figure5J/KEAP1-3-5J/KEAP1-3-C+MAKER-3-C2.tif]

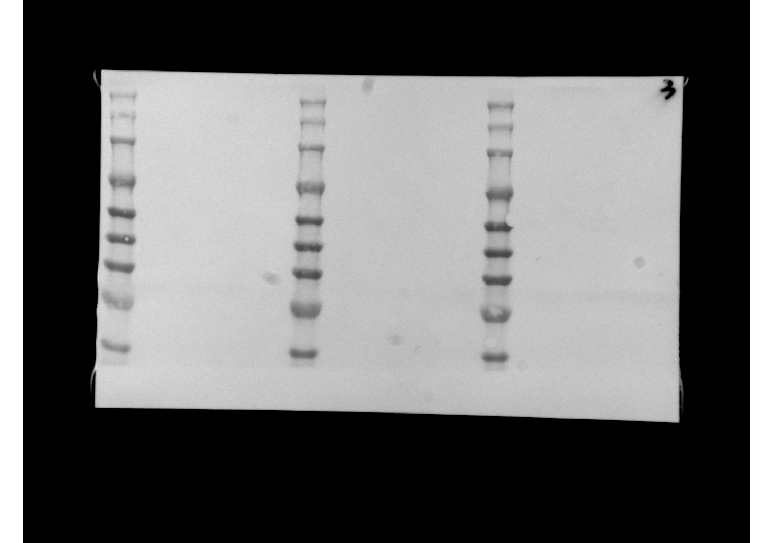

Supplement: Supplementary file 1 [file DataSheet_1.zip › Supplementary data/Figure5J/KEAP1-3-5J/MAKER-3-C.tif]

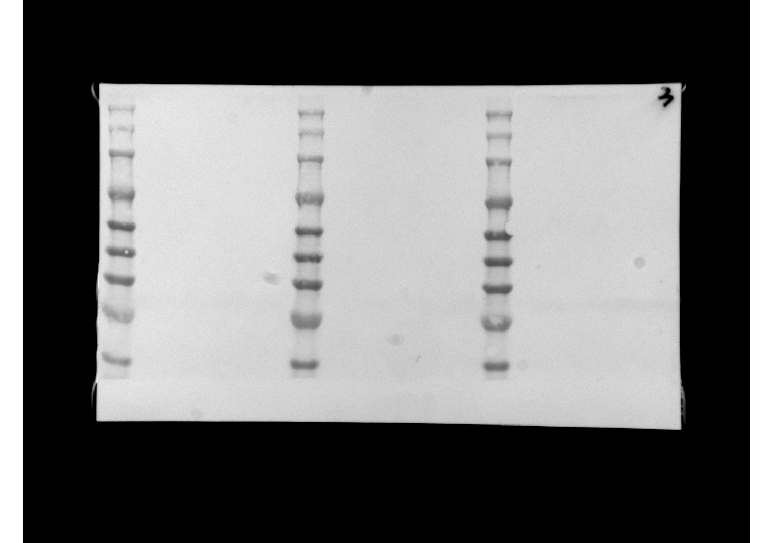

Supplement: Supplementary file 1 [file DataSheet_1.zip › Supplementary data/Figure5J/KEAP1-3-5J/MAKER-3-C2.tif]

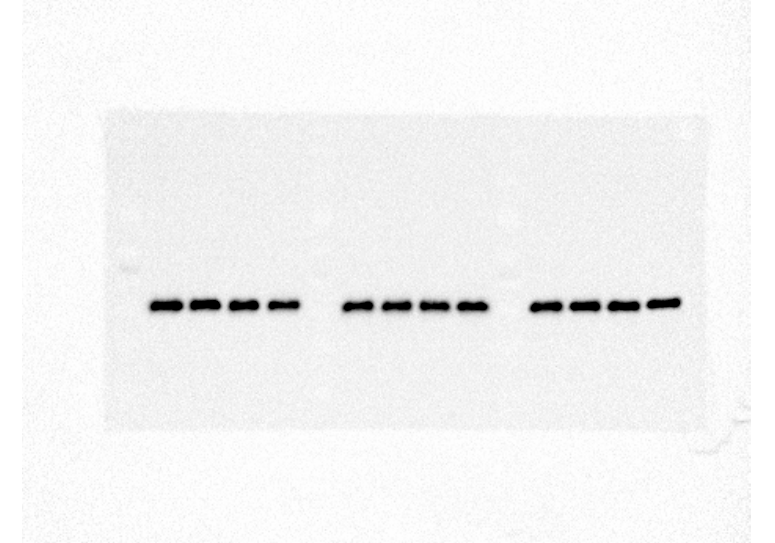

Supplement: Supplementary file 2 [file DataSheet_2.zip › Supplementary data-2/Figure4G/GAPDH-2-D/GAPDH-2-D.tif]

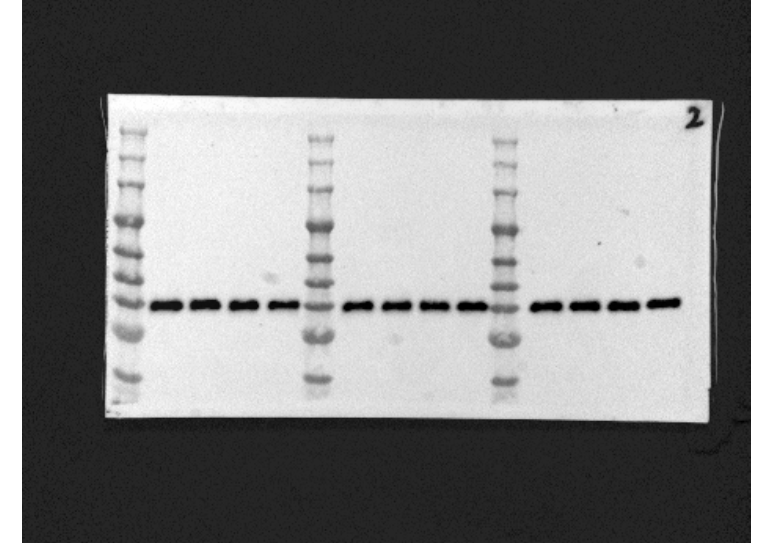

Supplement: Supplementary file 2 [file DataSheet_2.zip › Supplementary data-2/Figure4G/GAPDH-2-D/MAKER-2-D-K+GAPDH-2-D.tif]

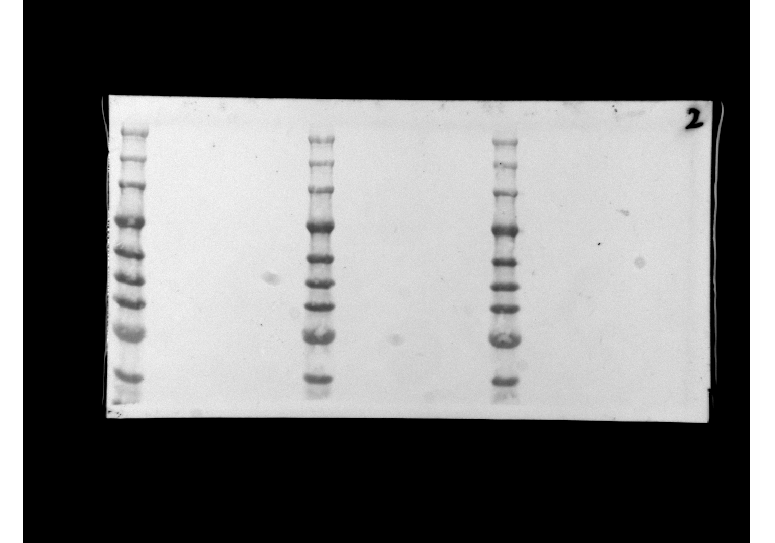

Supplement: Supplementary file 2 [file DataSheet_2.zip › Supplementary data-2/Figure4G/GAPDH-2-D/MAKER-2-D-K.tif]

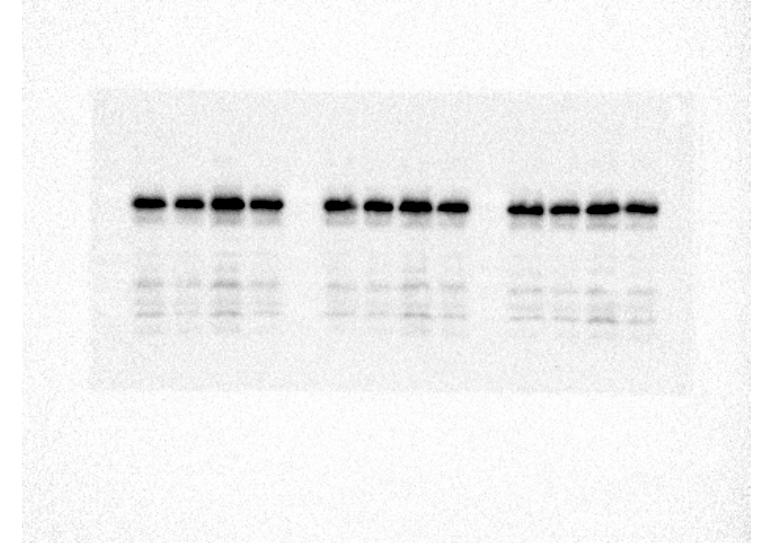

Supplement: Supplementary file 2 [file DataSheet_2.zip › Supplementary data-2/Figure4G/KEAP1-2-D/KEAP1-2-D.tif]

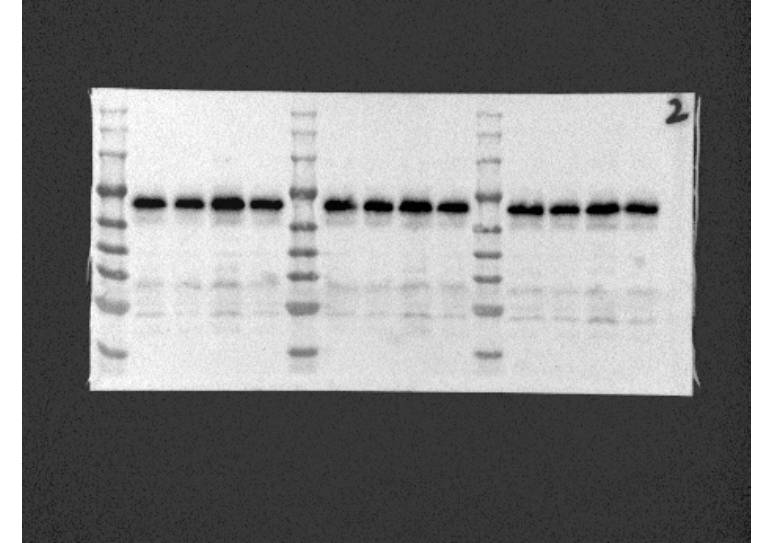

Supplement: Supplementary file 2 [file DataSheet_2.zip › Supplementary data-2/Figure4G/KEAP1-2-D/MAKER-2-K-D+KEAP1-2-D.tif]

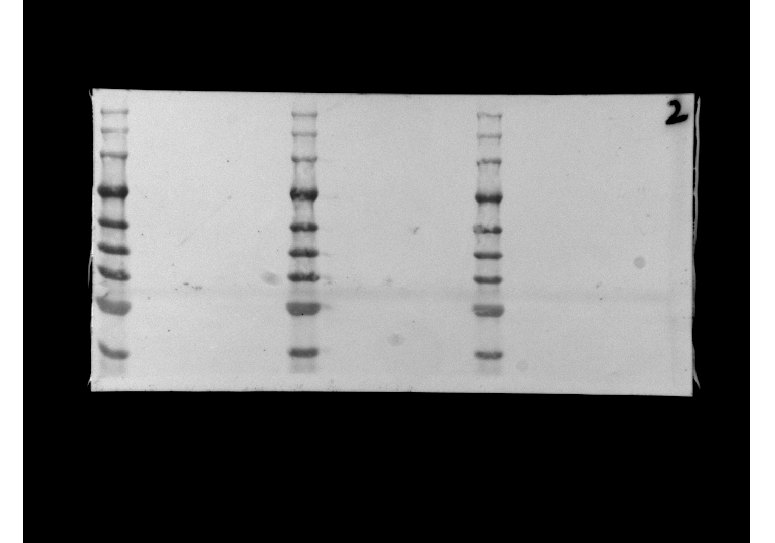

Supplement: Supplementary file 2 [file DataSheet_2.zip › Supplementary data-2/Figure4G/KEAP1-2-D/MAKER-2-K-D.tif]

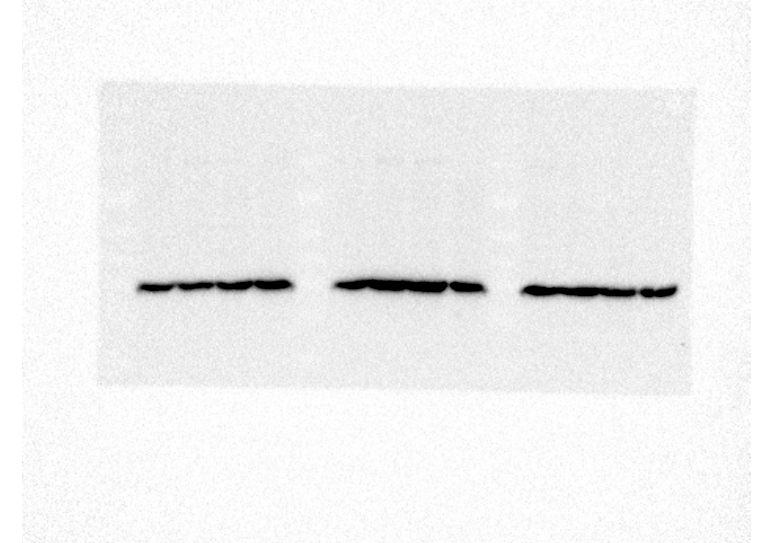

Supplement: Supplementary file 2 [file DataSheet_2.zip › Supplementary data-2/Figure4G-2/GAPDH-4-D/GAPDH-4-D.tif]

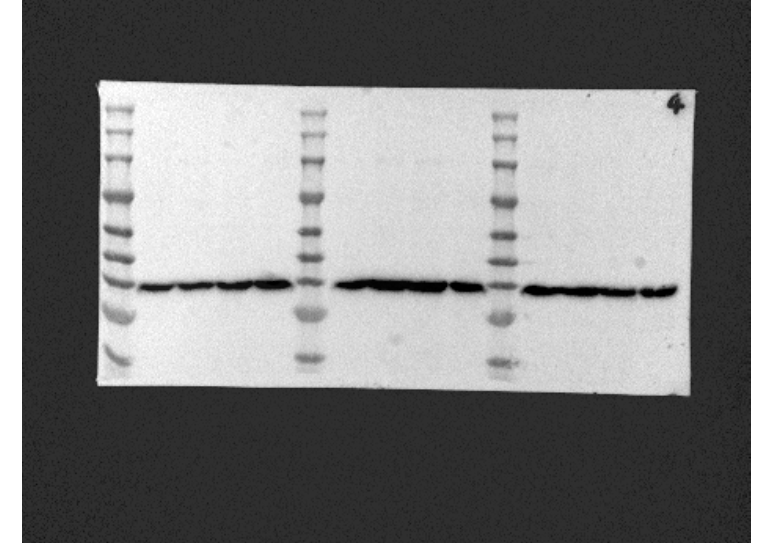

Supplement: Supplementary file 2 [file DataSheet_2.zip › Supplementary data-2/Figure4G-2/GAPDH-4-D/MAKER-4-D+GAPDH-4-D.tif]

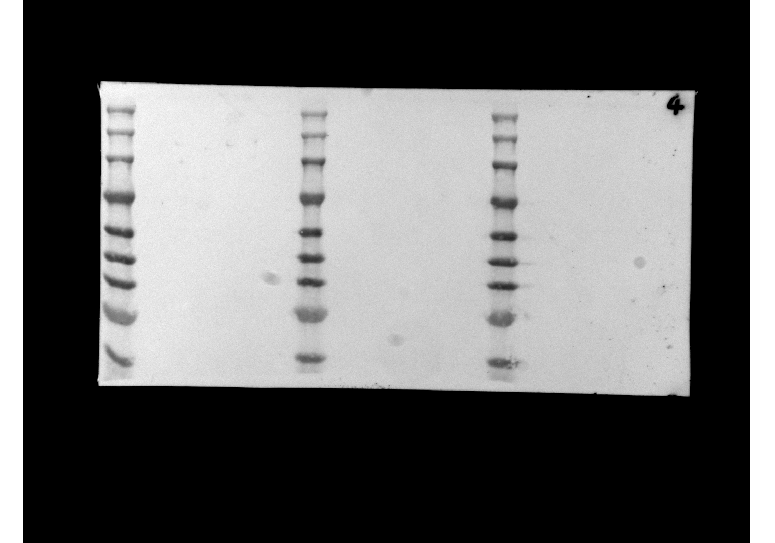

Supplement: Supplementary file 2 [file DataSheet_2.zip › Supplementary data-2/Figure4G-2/GAPDH-4-D/MAKER-4-D.tif]

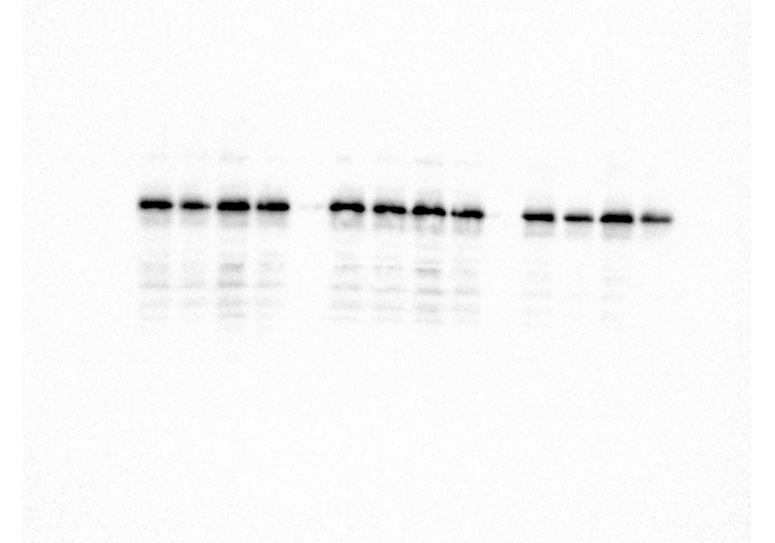

Supplement: Supplementary file 2 [file DataSheet_2.zip › Supplementary data-2/Figure4G-2/KEAP1-4-D/KEAP1-4-D.tif]

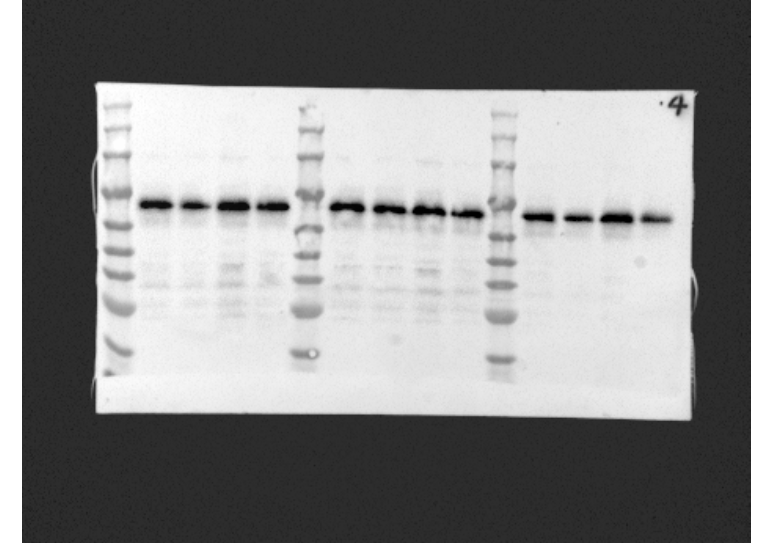

Supplement: Supplementary file 2 [file DataSheet_2.zip › Supplementary data-2/Figure4G-2/KEAP1-4-D/MAKER-4-K-D+KEAP1-4-D.tif]

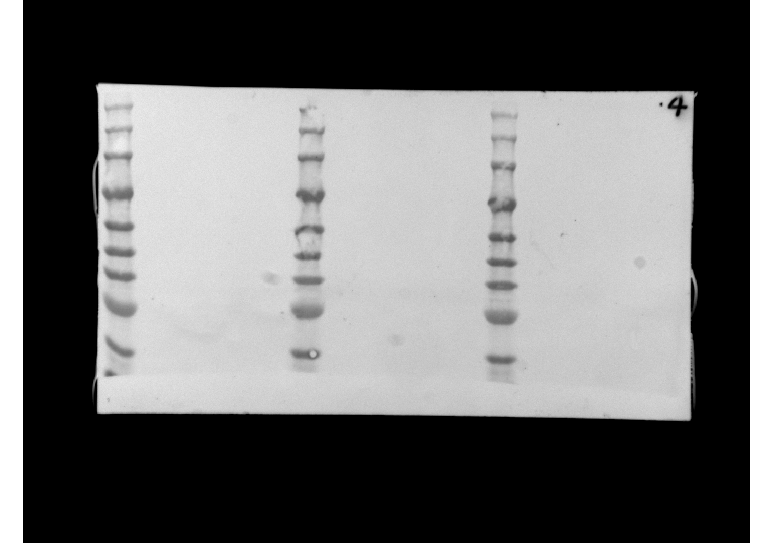

Supplement: Supplementary file 2 [file DataSheet_2.zip › Supplementary data-2/Figure4G-2/KEAP1-4-D/MAKER-4-K-D.tif]
