# Supplementary material for: miR-200a attenuated oxidative stress, inflammation, and apoptosis in dextran sulfate sodium-induced colitis through activation of Nrf2
Source: Front Immunol. 2023 Aug 14;14:1196065. doi: 10.3389/fimmu.2023.1196065 (PMC10461398; doi:10.3389/fimmu.2023.1196065)
Supplement: Supplementary file 2 [file DataSheet_2.zip › Supplementary data-2/Western blots Fig4G-Keap 1-GAPDH-23-6-29.pptx]

## Slide 1
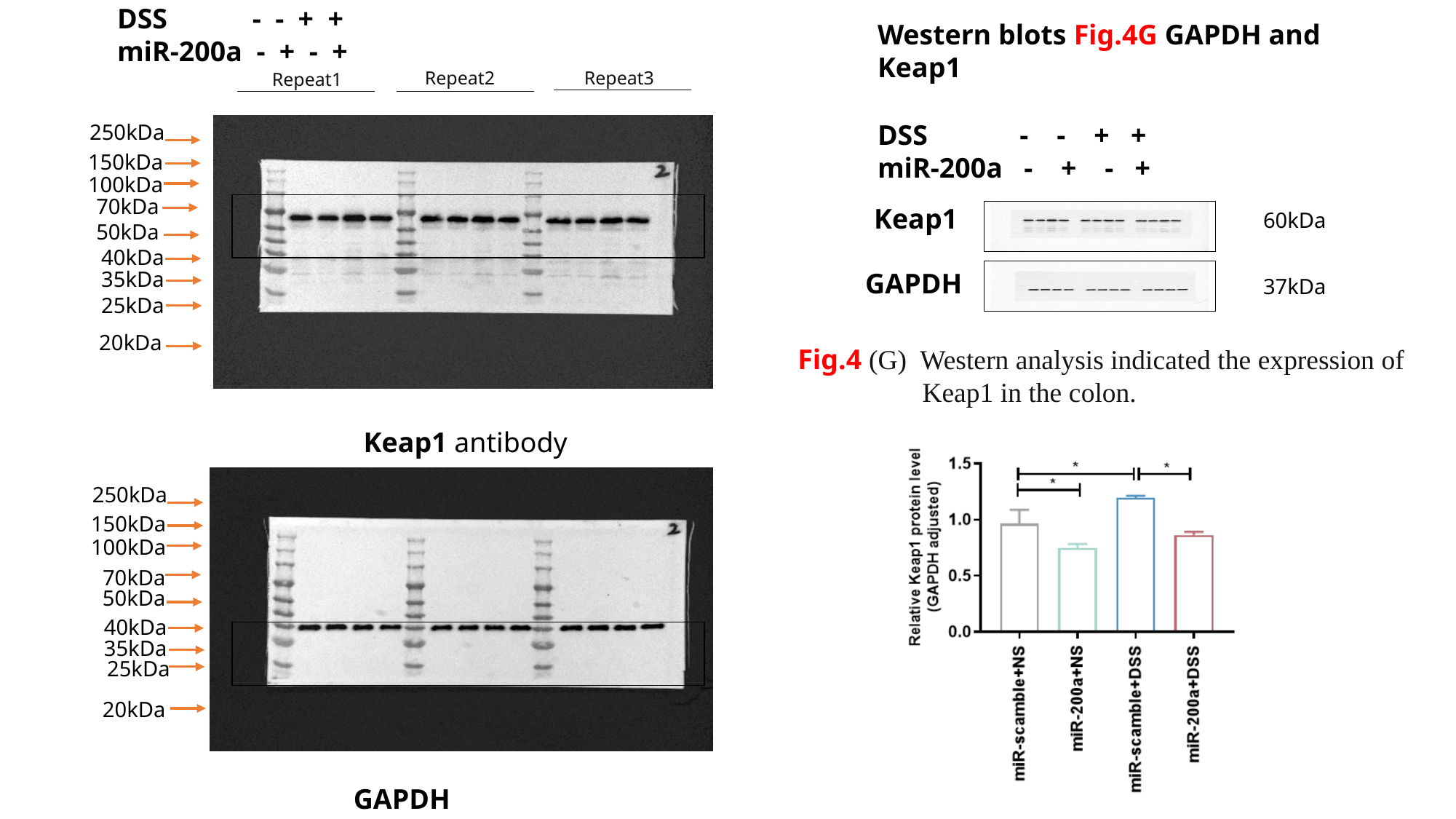

DSS - - + +
miR-200a - + - +
Western blots Fig.4G GAPDH and Keap1
Repeat2
Repeat3
Repeat1
DSS - - + +
miR-200a - + - +
250kDa
150kDa
100kDa
70kDa
Keap1
60kDa
50kDa
40kDa
35kDa
GAPDH
37kDa
25kDa
20kDa
Fig.4 (G) Western analysis indicated the expression of
 Keap1 in the colon.
Keap1 antibody
250kDa
150kDa
100kDa
70kDa
50kDa
40kDa
35kDa
25kDa
20kDa
GAPDH antibody

## Slide 2
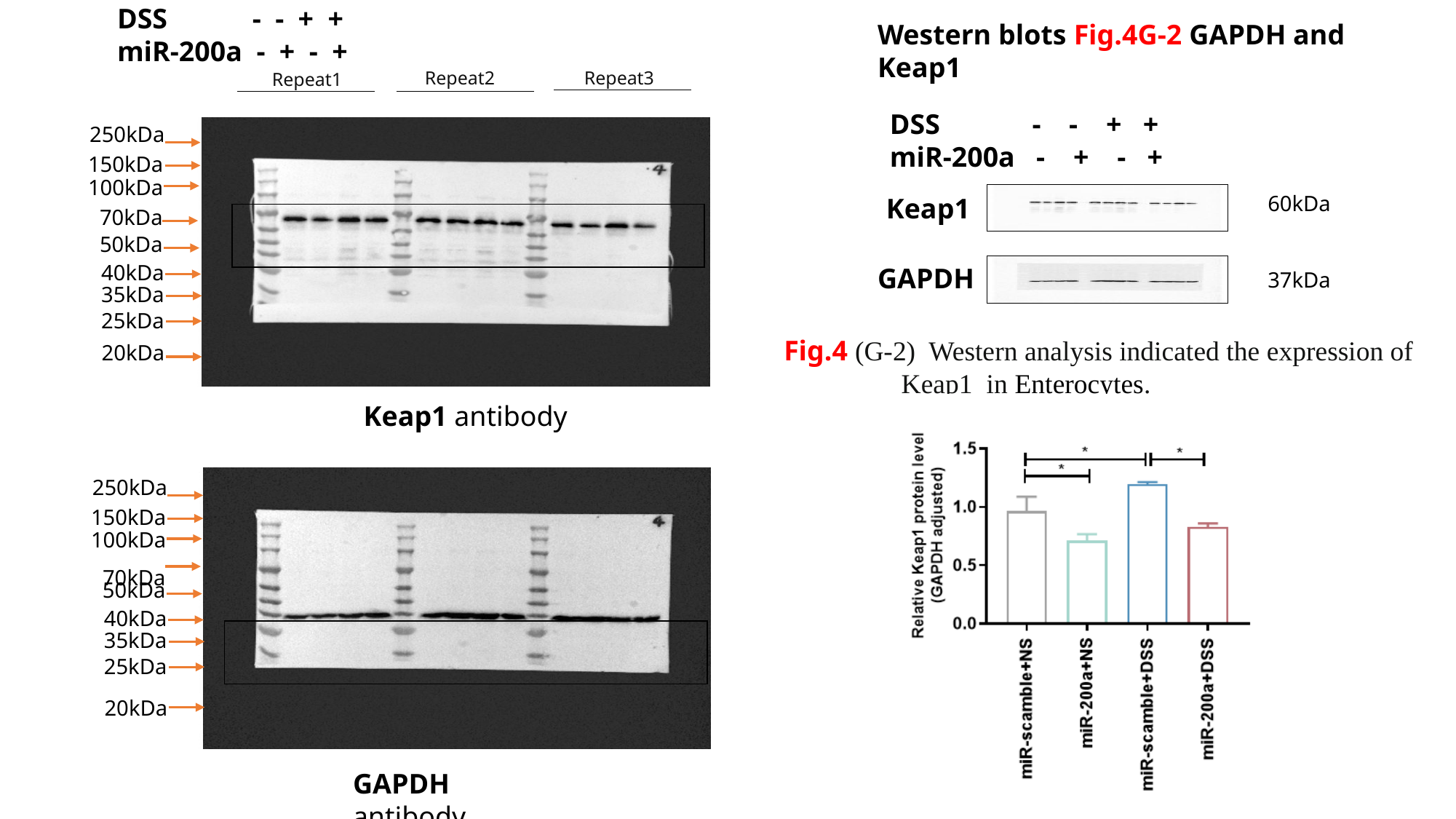

DSS - - + +
miR-200a - + - +
Western blots Fig.4G-2 GAPDH and Keap1
Repeat2
Repeat3
Repeat1
DSS - - + +
miR-200a - + - +
250kDa
150kDa
100kDa
60kDa
Keap1
70kDa
50kDa
40kDa
GAPDH
37kDa
35kDa
25kDa
Fig.4 (G-2) Western analysis indicated the expression of
 Keap1 in Enterocytes.
20kDa
Keap1 antibody
250kDa
150kDa
100kDa
70kDa
50kDa
40kDa
35kDa
25kDa
20kDa
GAPDH antibody

## Slide 3
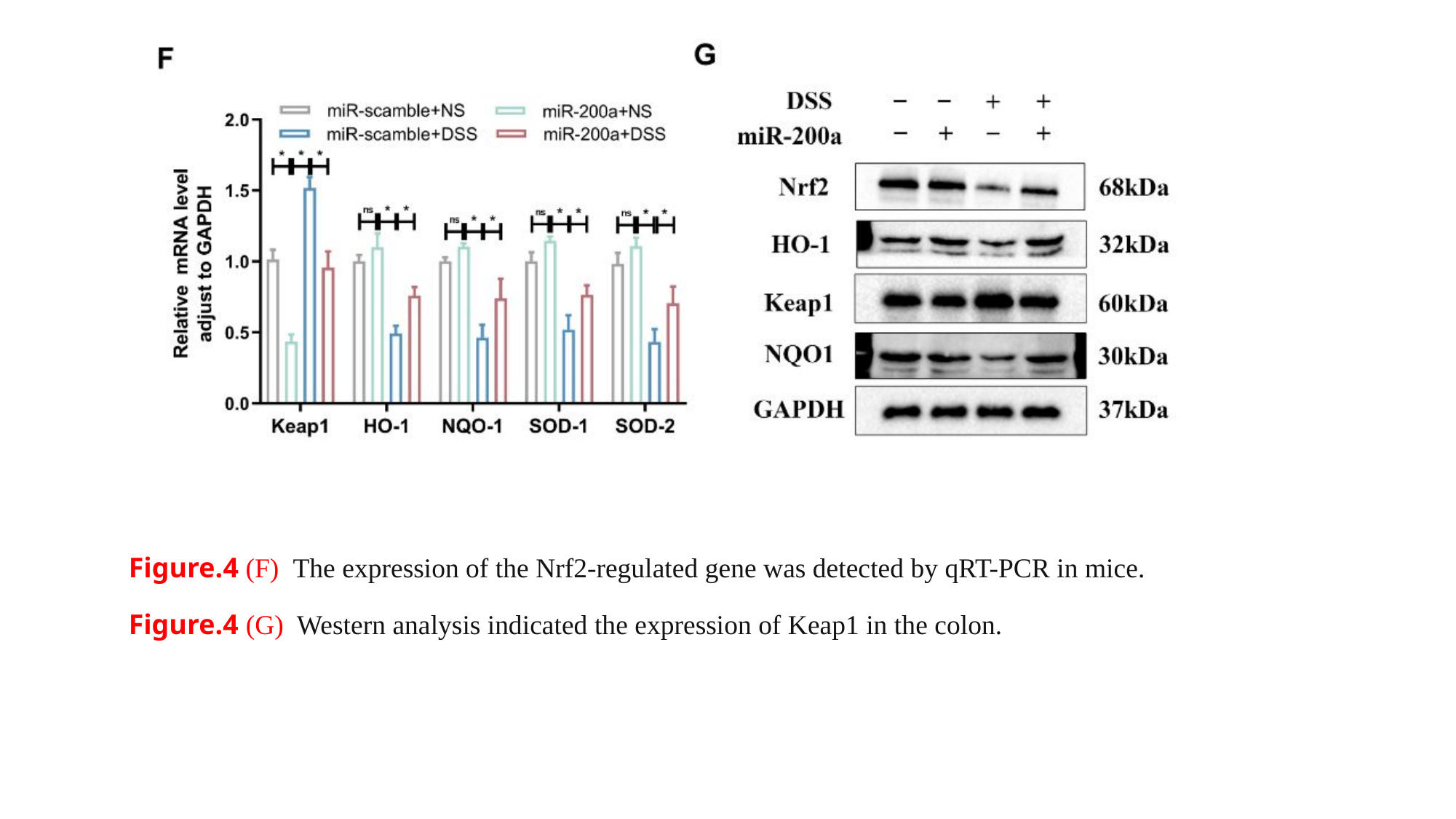

Figure.4 (F) The expression of the Nrf2-regulated gene was detected by qRT-PCR in mice.
Figure.4 (G) Western analysis indicated the expression of Keap1 in the colon.

## Slide 4
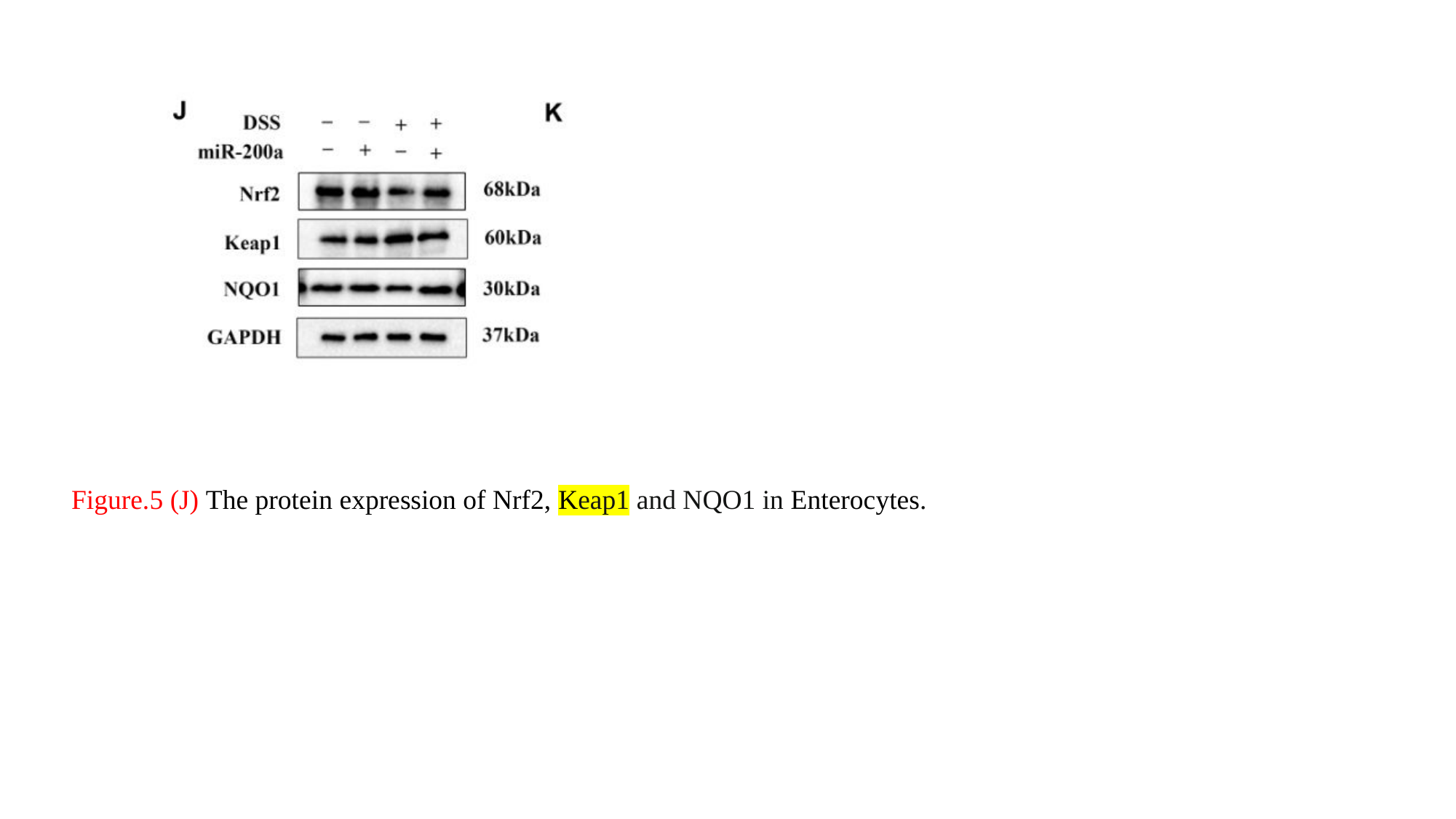

Figure.5 (J) The protein expression of Nrf2, Keap1 and NQO1 in Enterocytes.

## Slide 5
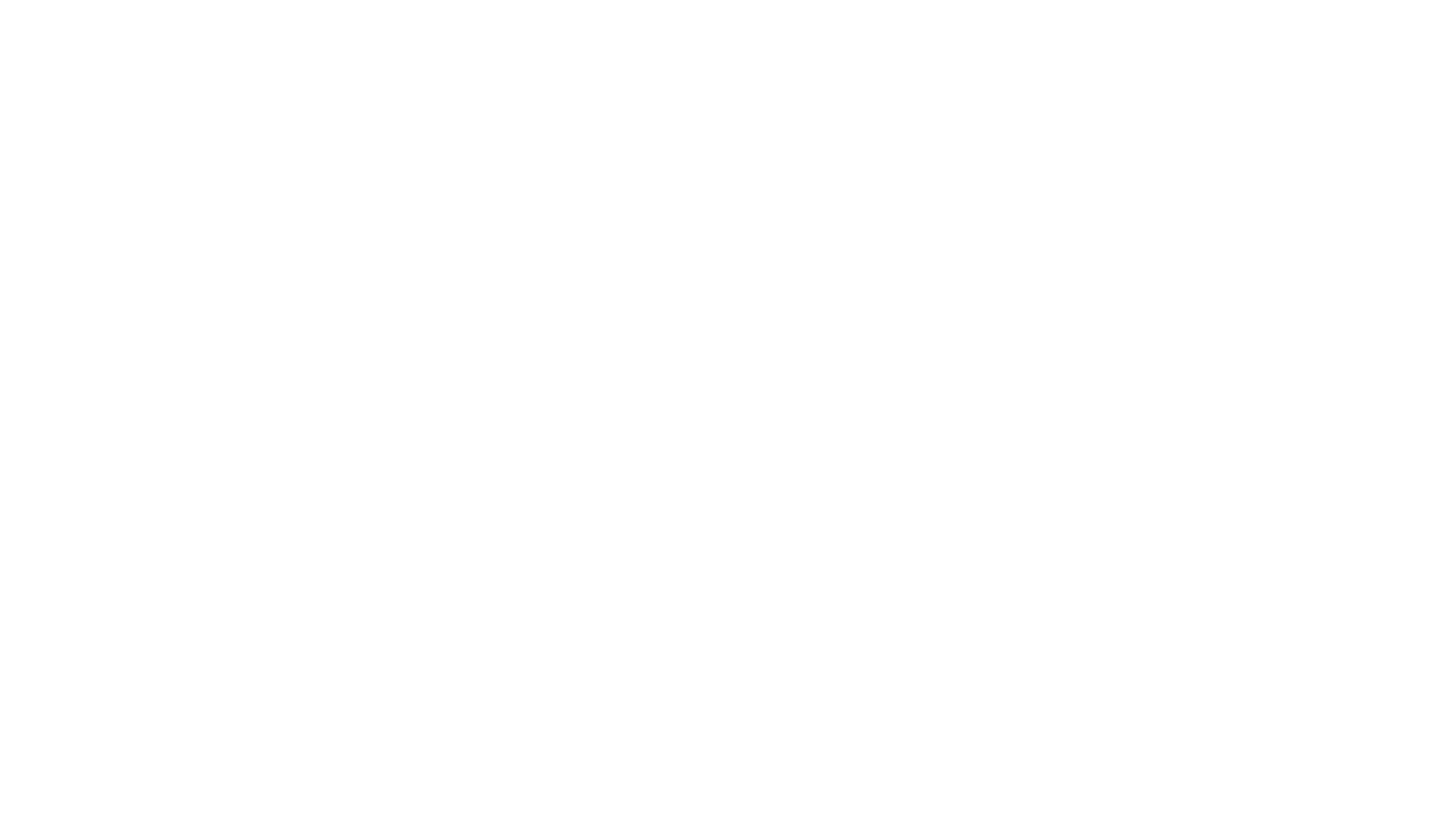

#
